# Role of traditional Chinese medicine monomers in cerebral ischemia/reperfusion injury:a review of the mechanism

**DOI:** 10.3389/fphar.2023.1220862

**Published:** 2023-08-16

**Authors:** Ting Zheng, Taotao Jiang, Zilong Huang, Hongxiang Ma, Manxia Wang

**Affiliations:** Department of Neurology, Lanzhou University Second Hospital, Lanzhou, China

**Keywords:** cerebral ischemia/reperfusion injury (CIRI), traditional Chinese medicine monomers, inflammation, programmed cell death, protective mechanism

## Abstract

Ischemia/reperfusion (I/R) injury is a pathological process wherein reperfusion of an ischemic organ or tissue exacerbates the injury, posing a significant health threat and economic burden to patients and their families. I/R triggers a multitude of physiological and pathological events, such as inflammatory responses, oxidative stress, neuronal cell death, and disruption of the blood-brain barrier (BBB). Hence, the development of effective therapeutic strategies targeting the pathological processes resulting from I/R is crucial for the rehabilitation and long-term enhancement of the quality of life in patients with cerebral ischemia/reperfusion injury (CIRI). Traditional Chinese medicine (TCM) monomers refer to bioactive compounds extracted from Chinese herbal medicine, possessing anti-inflammatory and antioxidative effects, and the ability to modulate programmed cell death (PCD). TCM monomers have emerged as promising candidates for the treatment of CIRI and its subsequent complications. Preclinical studies have demonstrated that TCM monomers can enhance the recovery of neurological function following CIRI by mitigating oxidative stress, suppressing inflammatory responses, reducing neuronal cell death and functional impairment, as well as minimizing cerebral infarction volume. The neuroprotective effects of TCM monomers on CIRI have been extensively investigated, and a comprehensive understanding of their mechanisms can pave the way for novel approaches to I/R treatment. This review aims to update and summarize evidence of the protective effects of TCMs in CIRI, with a focus on their role in modulating oxidative stress, inflammation, PCD, glutamate excitotoxicity, Ca^2+^ overload, as well as promoting blood-brain barrier repairment and angiogenesis. The main objective is to underscore the significant contribution of TCM monomers in alleviating CIRI.

## 1 Introduction

**FIGURE 1:**
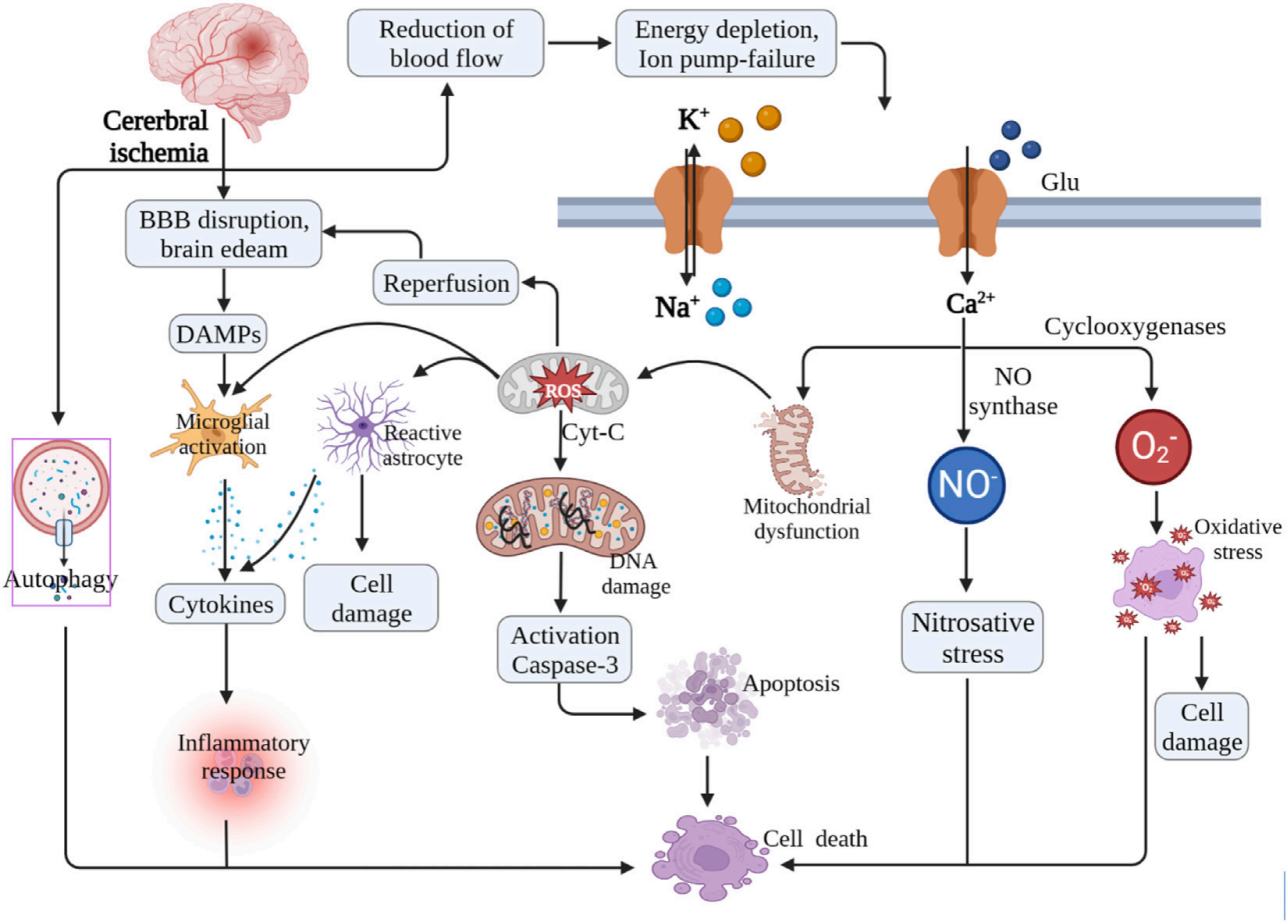
Mechanism of cerebral ischemic/reperfusion injury.

Ischemic stroke, the most prevalent acute cerebrovascular disease worldwide, is typically caused by a temporary or permanent reduction in cerebral blood flow resulting from thrombosis or thromboembolic artery occlusion (Roth et al., 2020). Thrombolytic therapy, aimed at restoring cerebral perfusion in a timely manner, is the main treatment strategy for ischemic brain injury (Stoll et al., 2008). However, reperfusion can potentially promote secondary cell death and exacerbate brain injury, leading to cerebral ischemia/reperfusion injury (CIRI) (Datta et al., 2020; Li et al., 2022a). The pathophysiology and pathogenesis of CIRI are complex and multifaceted, involving mitochondrial disorders, increased oxidative stress/reactive oxygen species (ROS), destruction of the blood-brain barrier (BBB), heightened inflammatory response and cell death (Leech et al., 2019; Ojo et al., 2019) (Figure 1). Current treatment for CIRI mainly includes thrombolytic therapy and conservative symptomatic therapy (such as drug and physical therapy). Although these traditional approaches can alleviate clinical symptoms to some extent, they rarely provide a cure by inhibiting the progression of the disease (Derex and Cho, 2017). The challenge in drug development stems from the intricate nature of CIRI’s pathological processes, which encompass pathological changes driven by factors that are not fully understood, including programmed cell death (PCD), oxidative stress and inflammatory responses. These pathological changes not only exert independent roles but also interact with each other, collectively accelerating CIRI. Therefore, it is imperative to explore novel drugs that target the underlying pathological progression of CIRI, with the goal of enhancing neurological recovery and prognosis in patients.

In recent years, traditional Chinese medicine (TCM) including herbs, formulas and monomers has gained considerable attention as an alternative and effective treatment for CIRI (Zhang et al., 2020a). TCM monomers, isolated from Chinese herbal medicine, are active substances with definite molecular formula and spatial structure (Wang et al., 2021a). They usually have specific pharmacological effects and targets and can be used for the treatment or prevention of CIRI. With the deepening understanding of TCM in recent years, some TCM monomers have demonstrated neuroprotective effects in CIRI (Wu et al., 2010). Compared with conventional therapy, TCM monomers possess unique and novel pharmacological mechanism advantages, allowing them to achieve similar therapeutic outcomes with reduced toxicity and side effects (Liu et al., 2020). Some beneficial TCM monomers, such as polyphenols, curcumin and puerarin, etc., have shown protective abilities in various animal models of nervous system diseases. Multiple experimental evidence has shown that TCM monomers may be effective in treating CIRI due to their antioxidant, free radical scavenging, anti-thrombotic and neuroprotective properties (Jivad and Rabiei, 2015; Ghandadi and Sahebkar, 2017; Mollazadeh et al., 2019). Furthermore, these TCM monomers can modulate multiple signaling pathways, influencing various pathophysiological processes of CIRI and alleviating its detrimental effects (Kovalska et al., 2012). Therefore, TCM monomers represent a crucial area of focus in the field of CIRI therapy in the future. In this review we arm to summarize the evidence the protective effects of TCM monomers on CIRI through diverse mechanisms. To elaborate the mechanism of TCM monomers in CIRI, we sorted out the relevant literature from 2013 to 2023 using databases sush as PubMed, Web of Science, CNKI, etc., with keywords such as “traditional Chinese medicine,” “cerebral ischemia-reperfusion injury,” “CIRI,” “mechanism,” etc. We screened 101 articles and identified 69 TCM monomers. The criteria for screening the literature: 1) based on an animal experiment, no restriction on animal species, gender, age, weight, and sample size; 2) involve a focal cerebral I/R damage model, caused by transient middle cerebral artery occlusion or middle cerebral artery occlusion/reperfusion (MCAO/R); 3) the experimental group was treated with only one traditional Chinese medicine monomer with specific dose or dose range but in no restriction on mode and time of initial treatment; 4) the control group was administered by saline, vehicle, or positive control drug or no treatment; 5) have one of the following outcomes available: infarct volume, neurological function score (NFS), and biochemical examinations; 6)including the specific and detailed mechanism of CIRI. Exclusion Criteria: The following exclusion criteria were also prespecified: 1) reviews, comments, case reports, editorials, clinical articles, and *in vitro* studies; 2) nonfocal brain I/R model, adopting global models (e.g., bilateral common carotid occlusion), traumatic models, or only hypoxic ischemic models; 3) absence of control group 4) outcome measures are not included in the literature; and 5) duplicated publications.

## 2 Protective mechanism of traditional Chinese medicine monomers in CIRI

**FIGURE 2:**
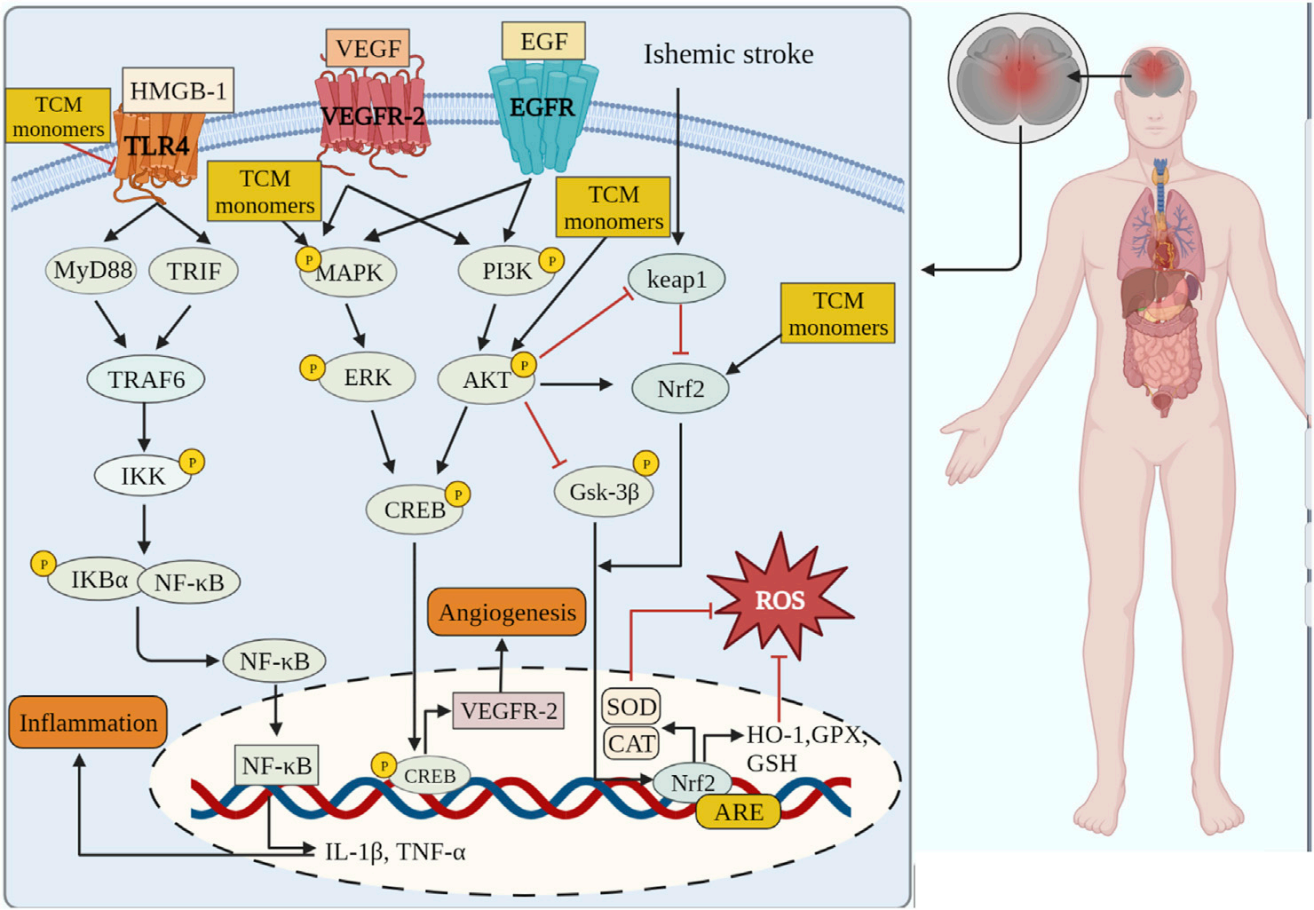
TCM monomers can play anti-inflammatory, antioxidant stress and promote angiogenesis in CIRI through related signaling pathways.

CIRI is a complex and dynamic process characterized by a series of mechanisms, including initial injury during the early stages of ischemia and subsequent injury following reperfusion. Insufficient blood flow during cerebral ischemia results in inadequate supply of glucose, oxygen, and excessive glutamate excitatory toxicity (Yang et al., 2018). The excessive release of glutamate can disrupt ion balance within cells, leading to Ca^2+^ overload (Kaviarasi et al., 2019). Ca^2+^ overload leads to the release of free radicals and NO, causing mitochondrial dysfunction and DNA damage, ultimately resulting in oxidative stress, neurotoxicity and inflammation of excitatory amino acids, and ultimately lead to cell death including necrosis, apoptosis and other forms of PCD and neurological disorders (Thiebaut et al., 2019) (Figure 2). TCM monomers are regarded as potential therapeutic agents for improving CIRI, reducing oxidative stress, inflammation and PCD, and modulating the pathological processes of CIRI through diverse mechanisms.

### 2.1 Effect caused by anti-oxidative stress

Oxidative stress plays a crucial role in the development of CIRI, leading to dysregulation of antioxidant defense system, cellular toxicity, damage and apoptosis (Liu et al., 2015). The imbalance between reactive oxygen species (ROS)/reactive nitrogen species (RNS) production and endogenous antioxidant defense mechanisms is the underlying pathogenesis of CIRI (Li et al., 2018a). Endogenous antioxidant defense systems, including superoxide dismutase (SOD), catalase (CAT), glutathione (GSH), and glutathione peroxidase (GSH-PX), deal with oxidant clearance and reduce oxidant-mediated brain damage. However, during CIRI, when the production of ROS/RNS exceeds the clearance capacity of the antioxidant defense system, oxidative stress is triggered, resulting in the release of numerous free radicals, lipid peroxidation, cell membrane damage, mitochondrial membrane destruction, and the exacerbation of cell injury and apoptosis (Perluigi et al., 2012; Sies, 2015). Moreover, oxidative stress during CIRI can directly induce DNA damage, activating pro-death signaling pathways and accelerating the apoptosis or necrosis of nervous system cells, thus impairing the recovery of neurological function (Li et al., 2018b). The reduction of oxidative stress through exogenous antioxidants is necessary in the context of CIRI.

Research conducted on a rat model of middle cerebral artery occlusion/reperfusion (MCAO/R) has demonstrated increased oxidative stress in the rat brain following injury. This is evident from the decrease in SOD activity, increase in lactate dehydrogenase (LDH) activity, and elevated levels of oxidative stress markers such as malondialdehyde (MDA), nitric oxide (NO), and neuronal nitric oxide synthase (iNOS) (Meng et al., 2018). A large number of TCM monomers have the effect of anti-oxidative stress and could show good antioxidant activity in both *in vivo* and *in vitro* CIRI models (Xu et al., 2020; Zeng et al., 2022). Studies have found that glycosides could act as exogenous antioxidants and promote the activity of antioxidant enzymes. For instance, Ginsenosides have been shown to inhibit ROS production and enhance the activities of CAT, SOD2, and GSH-Px (Zhou et al., 2006). In the rat model of MCAO/R, treatment with Ginsenoside Rb1 (20 mg/kg) significantly reduced MDA levels and increased the activities of SOD2 and GSH-Px. In addition, Ginsenoside Rb1 could also reduce the expression of NADPH oxidase 1 (NOX1), NADPH oxidase 4 (NOX4) and iNOS, as well as the activity of NOS in MCAO/R mice (Chen et al., 2015). The precise molecular mechanism underlying the antioxidant effects of glycosides remains unclear despite previous studies. Flavonoid monomers, in addition to their antioxidant effects, have been found to reduce oxidative stress in the MCAO/R rat model. Chrysin, for example, increased SOD levels and inhibited MDA expression after 5 days of administering a dose of 50 mg/kg (Shang et al., 2023). Similarly, eriocitrin was observed to provide protection against CIRI. In a study, male Sprague-Dawley rats were orally administered different doses of eriocitrin for 7 consecutive days. The group receiving a dosage of 32 mg/kg showed a significant increase in SOD expression in brain tissue and reduced MDA and LDH levels (He et al., 2020). Furthermore, eriocitrin was found to inhibit the Nrf2/HO-1/NQO1/NF-κB signaling pathway, alleviating oxidative damage in CIRI rats. Several other TCM monomers have also exhibited protective effects against CIRI by inhibiting oxidative stress, such as arjunolic acid (Yaidikar and Thakur, 2015), berberine (Zhang et al., 2016a; Shou et al., 2022), salvianolic acid A (Mahmood et al., 2017) and paeoniflorin (Wu et al., 2020).

Nuclear factor erythroid 2-related factor 2 (Nrf2) is a redox-sensitive transcription factor, when activated, interacts with antioxidant response elements to mediate antioxidant effects (Chen and Hsieh, 2020). Previous studies have shown that natural products or Chinese herbs exert a neuroprotective role against cerebral ischemia by activating the Nrf2/heme oxygenase 1 (HO-1) pathway. Thymoquinone, a phenolic monomer, has been found to regulate Nrf2 signaling in disease treatment, exerting a neuroprotective role and reducing dopaminergic neurodegeneration through Nrf2 signaling activation and subsequent alleviation of oxidative stress (Dong et al., 2020). Geraniin, a polyphenol TCM monomer, possesses antioxidant, anti-inflammatory, and antithrombotic biological activities. Male MCAO/R rats treated with intraperitoneal injections of Geraniin for 3 consecutive days at dosages of 5, 10, and 20 mg/kg·d exhibited significantly inhibited oxidative stress through Nrf2 activation, leading to increased SOD activity, reduced MDA and NO levels, and decreased infarct volume in CIRI. The concentration of 20 mg/kg·d showed the most prominent effects (Yang et al., 2022a). Britanin, an effective Nrf2 system inducer, inhibited the oxidative stress process by activating the Nrf2 protective pathway in the MCAO-R rat model (Wu et al., 2017a). However, in the CIRI process, the TCM monomers did not play the antioxidant role solely through the activation of Nrf2. Nrf2 could act in combination with signaling pathways such as Akt and MAPK. Akt is another upstream mediator of Nrf2, and knocking down of Akt can exacerbate brain I/R injury (Wu et al., 2021a). Neohesperidin, pre-administered via intraperitoneal injections once a day for 21 consecutive days at dosages of 10 mg/kg, 20 mg/kg, and 40 mg/kg, significantly upregulated SOD, GSH-PX and CAT activity, reduced MDA and MPO levels and increased protein expression of HO-1 in the MCAO/R rat model, thus preventing oxidative stress. The elevation of antioxidants was achieved through the Akt/Nrf2 signaling pathway, thereby protecting neurons from oxidative stress (Wang and Cui, 2013). A similar mechanism also has been reported by puerarin (Zhang et al., 2023). Emodin, on the other hand, can induce Nrf2 signal transduction through upregulation of AMP-activated protein kinase (AMPK), thereby preventing inflammation and exerting antioxidant activity (de Oliveira et al., 2021) (Table 1).

**TABLE 1:** Effects and mechanisms of different TCM monomers on oxidative stress in cerebral ischemia reperfusion injury.

TCM monomers	CAS No.	Molecular formula	Method of model	Mechanisms	Effects	References
**Ginsenoside Re**	52286-59-6	C_48_H_82_O_18_	MCAO/R	ROS↓, MDA↓, SOD2↑, GSH-PX	Anti-oxidative stress	Zhou et al. (2006)
**Ginsenoside Rb1**	22427-39-0	C_42_H_72_O_14_	MCAO/R, ODG/R	SOD2↑, GPX4↑, NOX1,4↓	Anti-oxidative stress	Chen et al. (2015)
**Chrysin**	480-40-0	C_15_H_10_O_4_	MCAO/R	MDA↓, SOD2↑	Anti-oxidative stress	Shang et al. (2023)
**Eriocitrin**	13463-28-0	C_27_H_32_O_15_	MCAO/R	ROS↓, MDA↓, Nrf2/HO-1/NQO1/NF-κB↓	Anti-oxidative stress	He et al. (2020)
**Arjunolic acid**	465-00-9	C_30_H_48_O_5_	MCAO/R	ROS↓, MDA↓	Anti-oxidative stress	Yaidikar and Thakur (2015)
**Berberine**	633-65-8	C_20_H_18_ClNO_4_	MCAO/R, OGD/R	PI3K/AKT↑, ROS↓, MDA↓, SOD2↑, NQO1↑, Nrf1↑, Nrf2↑	Anti-oxidative stress and neuroprotection	Zhang et al. (2016a), Shou et al. (2022)
**Salvianolic acid A**	115939-25-8	C_36_H_30_O_16_	MCAO/R	AKT↑, eNOS↓, peroxynitrite↓	Anti-oxidative stress	Mahmood et al. (2017)
**Paeoniflorin**	23180-57-6	C_23_H_28_O_11_	MCAO/R	MDA↓, SOD2↑	Anti-oxidative stress	Wu et al. (2020)
**Geraniin**	60976-49-0	C_41_H_28_O_27_	MCAO/R	Nrf2↑, MDA↓, NO↓	Anti-oxidative stress	Yang et al. (2022a)
**Britanin**	33627-28-0	C_19_H_26_O_7_	MCAO/R, OGD/R	Nrf2↑, MDA↓, NO↓, SOD2↑	Anti-oxidative stress	Wu et al. (2017a)
**Neohesperidin**	20702-77-6	C_28_H_34_O_15_	MCAO/R	AKT/Nrf2↑, SOD↑, MPO↓, MDA↓,GSH-PX↑, CAT↑	Anti-oxidative stress	Wang and Cui (2013)
**Puerarin**	3681-99-0	C_21_H_20_O_9_	MCAO/R, OGD/R	AKT/Nrf2↑, SOD↑, GPX↑, ROS↓, MDA↓, SOD2↑, GSH-PX↑, CAT↑	Anti-oxidative stress	Zhang et al. (2023)
Emodin	518-82-1	C_15_H_10_O_5_	MCAO/R	AMPK/Nrf2↑, SOD↑, GPX↑	Anti-oxidative stress	de Oliveira et al. (2021)

### 2.2 Inhibition of inflammation

Inflammation is a pathological reaction of the body to infections or tissue damage, and an important pathological process leading to ischemic brain injury and neurological dysfunction (Jin et al., 2010). Following reperfusion in the ischemic area, circulating cells (neutrophils, monocytes, and macrophages) and tissue resident cells (microglia, astrocytes, and endothelial cells) gather and migrate to the ischemic brain tissue in response to chemokines and cell adhesion molecules. This accumulation of cells is activated by the release of danger-/damage-associated molecular patterns (DAMPs), leading to the production of pro-inflammatory mediators (Arvin et al., 1996; Anrather and Iadecola, 2016). Inflammatory mediators and DAMPs promote inflammation by promoting chemotaxis of circulating immune cells (Iadecola and Anrather, 2011).

Numerous TCM monomers have demonstrated the ability to reduce inflammation after CIRI by inhibiting the production of pro-inflammatory cytokines and promoting the production of anti-inflammatory cytokines. Pro-inflammatory cytokines such as IL-1β, IL-6, and TNF-α play a crucial role in the inflammatory response following CIRI. They can activate matrix metalloproteinases (MMPs), disrupt the integrity of the blood-brain barrier (BBB), and contribute to BBB injury and hemorrhagic transformation (Lambertsen et al., 2012; Yang et al., 2019a). In the MCAO/R model, pretreatment with ginsenoside Rg1 at varying doses (40 mg/kg/day) once a day for 5 days significantly reduced the expression of pro-inflammatory cytokines TNF-α, IL-1β, and IL-6. Additionally, it inhibited the nuclear translocation of NF-κB and the phosphorylation of IκBα (*p* < 0.01). Ginsenoside Rg1 also reduced infarct volume, improved neurological deficit scores, and ameliorated histological appearance (*p* < 0.05) (Wang et al., 2018; Zheng et al., 2019). In addition, in a different study using the MCAO/R rat models, treatment of berberine at 40 mg/kg was able to promote the recovery of motor function after focal cerebral ischemia by down-regulating pro-inflammatory cytokines and up-regulating anti-inflammatory cytokines at this concentration (Maleki et al., 2018). Phenolic monomers are also anti-inflammatory agents of cerebral ischemic injury. In a 2 h/22 h I/R rat model of MCAO, a dose of 200 mg/kg curcumin can reduce the expression of pro-inflammatory factors IL-6, TNF-α and iNOS by inhibiting the TLR4/p38/MAPK pathway, thereby inhibiting inflammatory response and improving brain injury and nerve function (Huang et al., 2018). Icariin (Zheng et al., 2022), diosgenin (Zhang et al., 2022), and syringin (Liu et al., 2022) administered in the MCAO rat model, improved brain injury and neurologic function by downregulating pro-inflammatory cytokines TNF-α, IL-1β, and IL-6 to inhibit the inflammatory response.

The TLR4 and NF-κB pathways are signaling pathways function to regulate inflammation, and their activation is often associated with inflammatory responses (Clausen et al., 2016; Cho et al., 2019). Recent studies have shown that the TLR4/NF-κB signaling pathway is involved in the destructive inflammatory process of brain I/R injury. Increased expression of TLR4 protein in the plasma membrane activates TNF receptor associated factor 6 (TRAF6)/NF-κB signaling through myeloid differentiation factor 88 (MyD88). Binding of TLR4 to MyD88 leads to the activation of TRAF6 and NF-κB (Tsakiri et al., 2008). Many TCM monomers have been shown to target the TLR4/NF-κB pathway involved in the treatment of CIRI inflammation. In a CIRI study, rats in the MCAO1h/R24 h model were pretreated with salvianolic acid B intravenously at a dosage of 30 mg/kg once a day for 5 days. It was found that salvianolic acid B exerted an anti-inflammatory and neuroprotective role by inhibiting the transcriptional activity of the TLR4/MyD88/TRAF6/NF-kB signaling pathway and reducing the pro-inflammatory cytokine response (IL-1β, IL-6, and TNF-α) (Wang et al., 2016). In addition, in another study, MCAO/R rats were treated with various concentrations (5, 10, and 20 mg/kg) of Daphnetin. It was found that Daphnetin at 20 mg/kg concentration could significantly reduce the overexpression of TNF-α, IL-1β and IL-6 through the TLR4/NF-κB signaling pathway, and alleviate apoptosis of nerve cells, thus exerting neuroprotective and anti-inflammatory effects (Liu et al., 2016a) (Figure 2.).In addition to the TCM monomers, casticin (Huang et al., 2021a), saikosaponin A (Wang and Yang, 2020), salvianolic acid D (Zhang et al., 2020b), schisandrin B (Fan et al., 2020) and Z-Guggulsterone (Liu et al., 2018) have all played neuroprotective and anti-inflammatory roles in CIRI by inhibiting TLR4/NF-κB (Table 2).

**TABLE 2:** Effects and mechanisms of anti-inflammatory responses of different TCM monomers in cerebral ischemia-reperfusion injury.

TCM monomers	CAS No.	Molecular formula	Method of model	Mechanisms	Effects	References
Ginsenoside Rg1	22427-39-0	C_42_H_72_O_14_	MCAO/R	TNF-α↓, IL-1β↓, IL-6↓, IL-10↑	Anti-inflammatory response	Wang et al. (2018), Zheng et al. (2019)
Berberine	633–65-8	C_20_H_18_ClNO_4_	MCAO/R	TNF-α↓, IL-1β↓, IL-6↓, IL-10↑	Anti-inflammatory response	Maleki et al. (2018)
Curcumin	458–37-7	C_21_H_20_O_6_	MCAO/R	TLR4↓, IL-1↓,p-Akt↑ and p-mTOR↑ LC3-II/LC3-I↓	Anti-inflammatory response and Inhibit autophagy	Huang et al. (2018)
Icariin	489–32-7	C_33_H_40_O_15_	MCAO/R	TNF-α↓, IL-1β↓, IL-6↓, IL-10↑	Anti-inflammatory response	Zheng et al. (2022)
Ginkgolide B	15291-77-7	C_20_H_24_O_10_	MCAO/R, OGD/R	TLR4/NF-κB↓, TNF-α↓, IL-1β↓, IL-6↓, IL-10↑	Anti-inflammatory	Liu et al. (2022)
Salvianolic acid B	121521-90-2	C_36_H_30_O_16_	MCAO/R OGD/R	TLR4/NF-κB↓, TNF-α↓, IL-1β↓, IL-6↓, VEGF↑	Anti-inflammatory and promote angiogenesis	Wang et al. (2016)
Daphnetin	486–35-1	C_9_H_6_O_4_	MCAO/R	TLR4/NF-κB↓, TNF-α↓	Anti-inflammatory	Liu et al. (2016a)
				IL-1β↓, IL-6↓		
Casticin	479–91-4	C_19_H_18_O_8_	MCAO/R	TLR4/NF-κB↓, TNF-α↓	Anti-inflammatory	Huang et al. (2021a)
				IL-1β↓, IL-6↓		
Saikosap-oninA	20736-09-8	C_42_H_68_O_13_	MCAO/R	TLR4/NF-κB↓, TNF-α↓	Anti-inflammatory	Wang and Yang (2020)
				IL-1β↓, IL-6↓		
Salvianolic acid D	142998-47-8	C_20_H_18_O_10_	MCAO/R	TLR4/NF-κB↓, TNF-α↓	Inhibits inflammation and apoptosis	Zhang et al. (2020b)
				IL-1β↓, IL-6↓, caspase-3↓		
Schisandrin B	61281-37-6	C_23_H_28_O_6_	MCAO/R	TLR4/NF-κB↓, TNF-α↓, IL-1β↓, IL-6↓	Anti-inflammatory	Fan et al. (2020)
Z-Guggulsterone	39025-23-5	C_21_H_28_O_2_	MCAO/R	TLR4/NF-κB↓, TNF-α↓, IL-1β↓, IL-6↓	Anti-inflammatory	Liu et al. (2018)

### 2.3 Inhibition of programmed cell death

**FIGURE 3:**
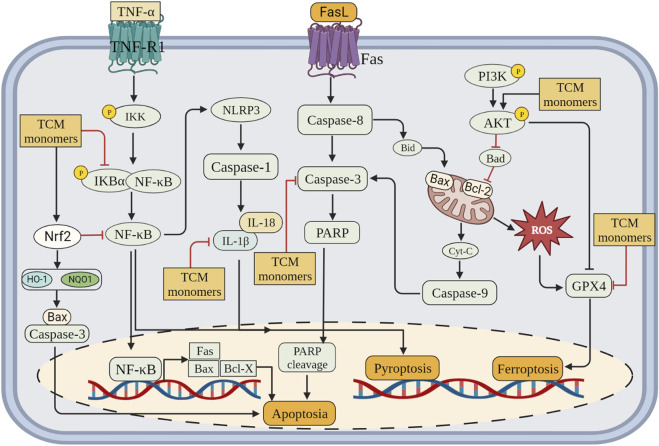
Inhibition of programmed cell death (apoptosis, pyroptosis and ferroptosis) by related TCM monomers in CIRI pathology.

CIRI is a series of processes, including cell death, that leads to deterioration of neurological function. CIRI has been reported to be associated with programmed cell death (PCD), including apoptosis, autophagy, pyroptosis and ferroptosis. Therefore, inhibition of PCD is very important for CIRI. Further investigations into TCM monomers have revealed their potential to inhibit PCD and provide protective effects in CIRI (Figure 3).

#### 2.3.1 Inhibition of apoptosis

Apoptosis is a regulated form of cell death characterized by cell contraction, cytoplasmic and nuclear condensation, and the formation of apoptotic bodies (Obeng, 2021). It is a unique gene-regulated process of cell death that is primarily triggered or accelerated during reperfusion or reoxygenation (Li and Jackson, 2002; Vanden Hoek et al., 2003). Various factors, such as disruption of calcium homeostasis, oxidative stress, DNA damage, and neuroinflammation, have been implicated in apoptosis induction (Broughton et al., 2009; Shin et al., 2018). Brain tissue ischemia leads to a decrease in intracellular ATP levels, resulting in mitochondrial dysfunction, translocation of Bax from the cytosol to the mitochondrial outer membrane, caspase activation, and initiation of apoptosis. Reperfusion not only amplifies this process but also provides the energy required for apoptosis (Eefting et al., 2004; Dorweiler et al., 2007). Cell apoptosis is a dynamic process, and the number of apoptotic cells increases with prolonged reperfusion time, further exacerbating brain injury (Zhang et al., 2016b). Apoptosis induced by CIRI is an important pathway of cell death, which can significantly aggravate neurological impairment in patients with cerebral infarction (Uzdensky, 2019). Therefore, timely intervention of cell apoptosis is beneficial to the prognosis of CIRI.

Caspase family members, including Caspase-3 and Caspase-8, are known to be essential factors regulating apoptosis (Kuranaga, 2012). Some TCM monomers can inhibit apoptosis by targeting Caspase-3, thereby exerting an anti-apoptotic protective role in ischemic brain injury (Chen et al., 2020). In MCAO/R rat models, baicalin and ginkgolide B inhibit cell apoptosis by increasing the Bcl-2/Bax ratio and decreasing caspase-3 levels (Yang et al., 2019b; Yang et al., 2021). Ligustrazine, a long-used therapeutic agent for ischemic cerebrovascular diseases, plays a significant role in preventing neuronal apoptosis by inhibiting the expression of CD62P, Sphk1, S1PR1, Bax/Bcl-2, and cleaved caspase-3 (Gao et al., 2015). In a mouse MCAO/R model with CIRI, ligustrazine targeted the circ_0008146/miR-709/Cx3cr1 axis to inhibit apoptosis after CIRI (Li et al., 2022b). TCM monomers have been reported to act on multiple targets within various signaling pathways, thereby alleviating CIRI-induced apoptosis. Polygalasaponin F promotes the expression of Bcl-2 and the Bcl-2/Bax ratio while inhibiting the expression of Bax and caspase-3 by activating the PI3K/Akt signaling pathway (Xie et al., 2020). In oxygen-glucose deprivation/reoxygenation (OGD/R)-treated primary cortical neurons, platycodin D decreases the expression of Bax but increases the expression of Bcl-2 by activating the PI3K/Akt signaling pathway (Wang et al., 2019a). Moreover, vitexin and triptolide inhibit cell apoptosis in MCAO rats by suppressing the c-Jun N-terminal kinase (JNK)/MAPK signaling pathway, leading to increased Bcl-2 expression and decreased Bax expression, ultimately reducing neurological deficits and neuronal damage (Wang et al., 2015a; Hao et al., 2015). Additionally, ginkgetin (Tian et al., 2019), dihydrocapsaicin (Wu et al., 2017b), carbenoxolone (Wang et al., 2015b), and genistein (Lu et al., 2019) activate the PI3K/Akt signaling pathway, inhibit apoptosis, and decrease the number of apoptotic cells.

#### 2.3.2 Regulation of autophagy

Autophagy is a cellular process whereby cells engulf cytoplasmic proteins or organelles, enclosing them into vesicles that fuse with lysosomes to form autophagosomes. These autophagosomes degrade their contents and contribute to the maintenance of organizational structure and function, promoting homeostasis under developmental and stress conditions (Shi et al., 2018). Appropriately regulated autophagy serves as a protective mechanism, playing a crucial role in cell survival and intracellular environment stability during stress states such as ischemia and hypoxia.

The beneficial or detrimental effects of autophagy on cerebral ischemia-reperfusion injury depend on the degree and timing of autophagic activity. Previous research has demonstrated that TCM monomers can modulate autophagy to ameliorate CIRI. For instance, ephedrine and galuteolin have been found to inhibit autophagy (Zhu et al., 2020; Shi et al., 2021). Vitexin, puerarin, and alphaasarone have been reported to reduce neuronal autophagy by decreasing Beclin1 expression and the LC3II/LC3I ratio, thus mitigating autophagic activity, while astragaloside IV and nicotiflorin could enhance autophagy and reduce neuronal apoptosis by increasing LC3II/LC3I ratio (Hongyun et al., 2017; Jiang et al., 2018; Zhang et al., 2019a; Zhang et al., 2021a; Wang et al., 2021b). In addition, TCM monomers preconditioning could also reduce autophagy in CIRI. Pre-administration of betulinic acid (50 mg/kg *in vivo*) could increase the expression of SIRT1, reduce the acetylation of FOXO1, and thus reduce the expression of autophagy protein (Zhao et al., 2021). Phillyrin (100 mg/kg *in vivo*, 80 μm *in vitro*) could reduce the expression of autophagy protein in MCAO/R animal models and H_2_O_2_-induced cell models, thereby reducing CIRI (Chen et al., 2022a). Autophagy has also been enhanced through pre-administration of berberine (40 mg/kg *in vivo*, 10^−5 ^μg/L *in vitro*) (Zhang et al., 2016c).

**FIGURE 4:**
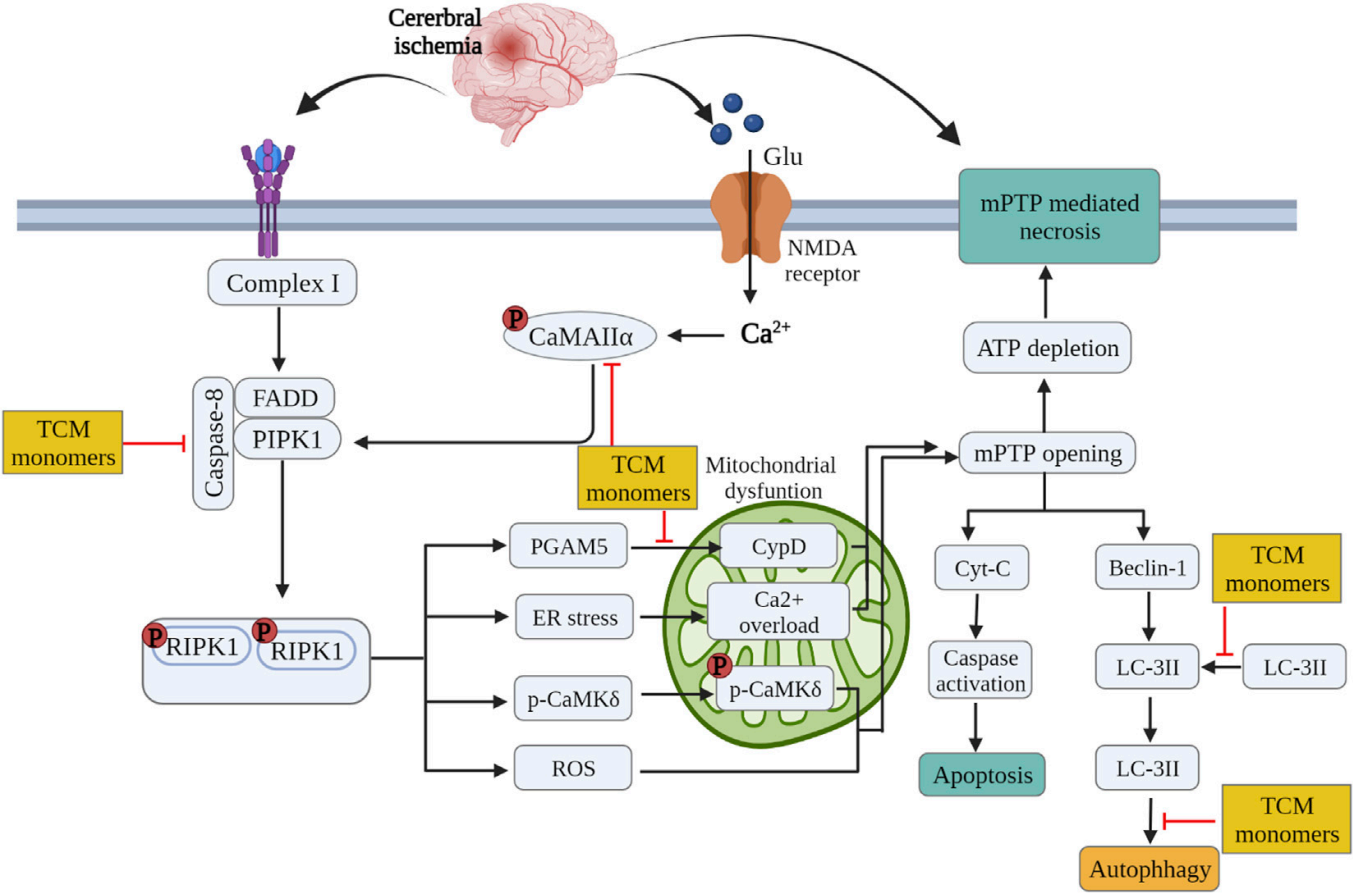
Inhibition of glutamate excitatory toxicity/Ca^2^+ overload, mitochondrial protection and regulation of autophagy by related TCM monomers in CIRI pathology.

TCM monomers modulate the process of autophagy by targeting various cellular signaling pathways. Deltonin (100 mg/kg) from *Dioscorea zingiberensis* C.H.Wright inhibited the expression of autophagy proteins Beclin-1, LC3II/I and p62 by directly reducing the expression of p-Akt and p-mTOR in MACO/R models (Zhang et al., 2020c). In addition, curcumin at a dose of 200 mg/kg can also reduce autophagy activity by inhibiting PI3K/Akt/mTOR signaling in the MCAO/R model (Huang et al., 2018). In MCAO/R rat model and OGD/R HT22 cell model, Eugenol derived from traditional Chinese medicine *Acorus gramineus* Aiton significantly increased AMPK phosphorylation, decreased phosphorylation of mTOR and P70S6Ks, enhances the expression of autophagy related proteins Beclin-1, LC3II/I and p62 (Sun et al., 2020). (Figure 4.)

#### 2.3.3 Inhibition of pyroptosis

Pyroptosis is a pro-inflammatory programmed cell death pathway that depends on the activation of inflammasomes and is driven by the binding of cytoplasmic sensor proteins to pathogen-associated molecular patterns (PAMPs) or DAMPs (Sapkota et al., 2017). The inflammatory body complex is typically composed of Nod-like receptors (NLRPs) containing the pyranoid domain, adaptor apoptosis-associated speckle like proteins (ASC) containing CARD, and caspase. The oligomerization of NLRP3 with ASC and procaspase 1 activates the NLRP3 inflammasome, leading to the cleavage of procaspase 1. The activated caspase-1 then cleaves pre-IL-1β and pre-IL-18, generating the mature forms of these proteins, namely, IL-1β and IL-18, respectively (Sapkota et al., 2017). At present, the research on the inhibition of pyroptosis by TCM monomers mainly focused on their role on the formation of NLRP3 inflammasomes.

TCM monomers have been found to reduce CIRI by inhibiting the activity of the NLRP3 inflammasome. Studies have demonstrated that baicalin could decrease infarct size in MCAO/R rats, suppress the activation of the NLRP3 inflammasome, and inhibit pyroptosis by reducing the expression of pyroptosis-associated proteins (ASC, cleaved caspase-1, IL-1β, and IL-18) (Zheng et al., 2021). In addition, ephedrine (40 mg/kg) inhibited the activation of NLRP3 inflammasome and decreased the expression of Caspase-1 and IL-1β by activating Akt/glycogen synthase kinase 3β(GSK3β)/Nrf2 pathway in MCAO/R mice, effectively inhibiting microglia pyroptosis and thus playing a neuroprotective role in CIRI (Li et al., 2021). In addition to baicalin and ephedrine, other TCM monomers have also been proven to play an anti-pyroptosis role by inhibiting the activation of NLRP3 inflammasome, such as ginsenoside Rd (Yao et al., 2022), sinomenine (Qiu et al., 2016), diosmetin (Shi et al., 2022). In addition, Chinese medicine monomers could inhibit NLRP3 inflammasome in CIRI to play an anti-pyroptotic role by regulating various signaling pathways, such as NF-κB, Nrf2, AMPK, and JAK2/STAT3 Signal Transducer and Activator of Transcription 3(STAT3) signaling pathway. The NF-κB pathway plays a central role in the aggregation of NLRP3 components and the formation of NLRP3 inflammasome activation (Hasanzadeh et al., 2020). For instance, curcumin and rubesin inhibited NF-κB signaling, thereby reducing NLRP3 inflammasome activity and exerting an anti-pyroptotic effect (Ran et al., 2021a; Jia et al., 2021). Nrf2 has been shown to negatively regulate NLRP3 inflammasome activation and plays an important role in CIRI. Astragaloside IV and ginsenoside Rd inhibit NLRP3 inflammasome-induced pyroptosis through Nrf2 modulation (Xiao et al., 2021; Yao et al., 2022). AMPK influences neuronal repair and angiogenesis in ischemic tissue during CIRI (Chen et al., 2019). Hispidulin and ephedrine exert their neuroprotective effects both *in vivo* and *in vitro* by modulating the AMPK/GSK-3β signaling pathway to alleviate NLRP3-mediated pyroptosis (An et al., 2019; Li et al., 2021). Sirtuin 1 (SIRT-1) activation is also important in neuroprotective mechanisms (Wang et al., 2019b). Tetrandrine and arctigenin have been found to inhibit the NLRP3 inflammasome by activating SIRT-1, thereby reducing pyroptotic cell injury in in vivo and *in vitro* experiments (Zhang et al., 2017; Wang et al., 2020b).

#### 2.3.4 Inhibition of ferroptosis

Ferroptosis is a recently identified form of cell death characterized by the iron-dependent accumulation of lipid-based reactive oxygen species (ROS). This process is regulated by the inactivation of glutathione peroxidase 4 (GPX4), which normally reduces lipid peroxides at the expense of glutathione (GSH). Ferroptosis exhibits three distinct features: 1) the accumulation of iron, which leads to the fenton chemical reaction, resulting in the release of large amounts of ROS; 2) The metabolism of certain amino acids was disordered, the expression of GPX4 and ferritin was increased, and the expression of acyl-CoA synthetase member 4 (ACSL4) was increased; 3) unlike apoptosis and necrosis, ferroptosis involves structural damage to mitochondria, including reduced mitochondrial volume, increased bilayer membrane density, and even the disappearance of mitochondrial cristae.

Guo et al. have shown that carthamin yellow could reduce the accumulation of ROS and free iron in the brain tissue of MCAO/R model animals, increased the levels of GPX4 and GSH, thus reducd neuronal injury and improving CIRI (Guo et al., 2021). In addition, β-Caryophyllene has similar effects to ferrostatin-1, an iron scavenger and ferroptosis inhibitor. β-Caryophyllene could inhibit hypoxia-induced iron overload, improve neuronal survival rate and upregulate GSH level, decrease ROS level, downregulate ACSL4 protein expression, ferritin and Gpx4 protein expression, and alleviate mitochondrial structure damage after OGD/R injury. The mechanism may be to improve the survival of astrocytes through Nrf2/HO-1 pathway, and thus reduced neurological function defects (Hu et al., 2022). In addition, other studies have found that rehmannioside A could reduce CIRI by activating PI3K/AKT/Nrf2 and SLC7A11/GPX4 pathway of ferroptosis (Fu et al., 2022). From the above studies, it is not difficult to find that TCM monomers could play a role in programmed cell death in CIRI through a variety of pathways (Table 3).

**TABLE 3:** Effects and mechanisms of different TCM monomers on programmed cell death in cerebral ischemia-reperfusion injury.

TCM monomers	CAS No.	Molecular formula	Method of model	Mechanisms	Effects	References
Baicalin	21967-41-9	C_21_H_18_O_11_	MCAO/R	NLRP3↓, IL-1β↓, IL-18↓, ASC↓, Bax↓, Bcl-2↑, caspase3↓	Anti-apoptosis and pyroptosis	Yang et al. (2019b), Zheng et al. (2021)
Ligustrazine	1124-11-4	C_8_H_12_N_2_	MCAO/R	Bax↓, Bcl-2↑, caspase-3↓	Anti-apoptosis	Gao et al. (2015), Li et al. (2022b)
Polygalasaponin F	882664-74-6	C_53_H_86_O_23_	MCAO/R OGD/R	PI3K/AKT↑, Bcl-2↑, Bax↓, caspase-3↓	Anti-apoptosis	Xie et al. (2020)
Platycodin D	58479-68-8	C_57_H_92_O_28_	MCAO/R	PI3K/AKT↑, Bcl-2↑, Bax↓	Anti-apoptosis	Wang et al. (2019a)
Vitexin	3681-93-4	C_21_H_20_O_10_	MCAO/R	JNK/MAPK↓, caspase-3↓ Bax↓, Bcl-2↑	Anti-apoptosis	Wang et al. (2015a)
Triptolide	38748-32-2	C_20_H_24_O_6_	MCAO/R	JNK/MAPK↓, Bcl-2↑, Bax↓	Anti-apoptosis	Hao et al. (2015)
Ginkgetin	481-46-9	C_32_H_22_O_10_	MCAO/R	PI3K/AKT↑, Bcl-2↑, Bax↓, caspase-3↓	Anti-apoptosis	Tian et al. (2019)
Dihydrocapsaicin	19408-84-5	C_18_H_29_NO_3_	MCAO/R	PI3K/AKT↑, Bcl-2↑, Bax↓, caspase-3↓	Anti-apoptosis	Wu et al. (2017b)
Carbenoxolone	7421-40-1	C_34_H_48_Na_2_O_7_	MCAO/R	PI3K/AKT↑, Bcl-2↑, Bax↓, caspase-3↓	Anti-apoptosis	Wang et al. (2015b)
Genistein	446-72-0	C_15_H_10_O_5_	MCAO/R	PI3K/AKT↑, Bcl-2↑, Bax↓, caspase-3↓	Anti-apoptosis	Lu et al. (2019)
Curcumin	458-37-7	C_21_H_20_O_6_	MCAO/R	p-Akt↑, p-mTOR↑, LC3-II/LC3-I↓	Inhibit autophagy	Huang et al. (2018)
Ephedrine	579-07-7	C_9_H_8_O_2_	MCAO/R	pNF-kB↓, Beclin-1↓, LC3 II ↓	Inhibit autophagy	Shi et al. (2021)
Galuteolin	20344-46-1	C_21_H_20_	MCAO/R	Beclin-1↓, LC3II/I↓, p62↑	Inhibit autophagy	Zhu et al. (2020)
Vitexin	3681-93-4	C_21_H_20_O_10_	MCAO/R	mTOR↑, Ulk1↓, PPAR-γ↑, Beclin1↓, p62↑, LC3Ⅱ↓	Inhibit autophagy	Jiang et al. (2018)
Puerarin	3681-99-0	C_21_H_20_O_9_	MCAO/R	LC3-II↓, LC3-II/LC3-I↓	Inhibit autophagy	Hongyun et al. (2017)
Alphaasarone	2883-98-9	C_12_H_16_O_3_	MCAO/R OGD/R	LC3-II/LC3-I↓, p62↑	Inhibit autophagy	Zhang et al. (2021a)
Betulinic acid	472-15-1	C_30_H_48_O	MCAO/R OGD/R	SIRT1↑, acetylated FoxO1↓, Beclin1↓, p62↑, LC3-II/LC3-I↓	Inhibit autophagy	Zhao et al. (2021)
Phillyrin	487-41-2	C_27_H_34_O_11_	MCAO/R	pAkt-1↑, pmTOR↑, Beclin1↓, LC3-II/LC3-I↓	Inhibit autophagy	Chen et al. (2022a)
Astragaloside IV	84687-43-4	C_41_H_68_O_14_	MCAO/R OGD/R	LC3-II/LC3-I↑, p62↓	Enhance autophagy	Zhang et al. (2019a)
Berberine	633-66-9	C_20_H_19_NO_8_S	MCAO/R OGD/R	Beclin1↓, p62↓, LC3-II/LC3-I↑	Enhance autophagy	Zhang et al. (2016c)
Nicotiflorin	17650-84-9	C_27_H_30_O_15_	MCAO/R OGD/R	p-mTOR↓, Beclin1↓, p62↓, LC3-II/LC3-I↑	Enhance autophagy	Wang et al. (2021b)
Deltonin	55659-75-1	C_45_H_72_O_17_	MCAO/R	p-Akt↑ and p-mTOR↑ LC3-II/LC3-I↓, Beclin-1↓	Inhibit autophagy	Qiu et al. (2016)
Eugenol	97-53-0	C_10_H_12_O_2_	MCAO/R, OGD/R	p-AMPK/AMPKα↑, p-mTOR/mTOR↓, pP70S6K/P70S6K↓,Beclin-1↑, LC3II/I↑, p62↓	Enhance autophagy	Shi et al. (2022)
Ephedrine	579-07-7	C_9_H_8_O_2_	MCAO/R	Akt/GSK3β/NRF2↑, IL-1β↓, NLRP4↓, IL-18↓	Inhibit pyroptosis	Zheng et al. (2021)
Sinomenine	115-53-7	C_19_H_23_NO_4_	MCAO/R, OGD/R	AMPK, NLRP4↓, IL-1β↓IL-18↓, IL-1β↓, caspase-1↓, TNF-α↓	Anti-inflammatory and inhibit pyroptosis	Yao et al. (2022)
Diosmetin	520-34-3	C_16_H_12_O_6_	MCAO/R, OGD/R	NLRP4↓, IL-18↓, IL-1β↓	Inhibit pyroptosis	Qiu et al. (2016)
Oridonin	28957-04-2	C_20_H_28_O_6_	MCAO/R OGD/R	NF-κB↓, NLRP4↓, IL-18↓, IL-1β↓	Inhibit pyroptosis	Jia et al. (2021)
Astragaloside IV	84687-43-4	C_41_H_68_O_14_	MCAO/R	Nrf2↓, NLRP4↓, IL-18↓, IL-1β↓, MMP4,9↓, TJs↑	Inhibit pyroptosis and maintain BBB	Xiao et al. (2021)
Hispidulin	1447-88-7	C_16_H_12_O_6_	MCAO/R	AMPK/GSK3β↓, NLRP4↓, IL-18↓, IL-1β↓	Inhibit pyroptosis	An et al. (2019)
Tetrandrine	518-34-3	C_38_H_42_N_2_O_6_	MCAO/R	Sirt-1↑, NLRP4↓, IL-18↓, IL-1β↓	Inhibit pyroptosis	Wang et al. (2020a)
Carthamin yellow	1401-20-3	C_21_H_22_O_11_	MCAO/R	NLRP3↓, NF-kB↓,ROS↓, Fe2+↓,GPX4↑,ACSL4↓,FTH1↑,TFR1↓	Inhibit ferroptosis	Guo et al. (2021)
β-Caryophyllene	87-44-5	C_15_H_24_	MCAO/R, OGD/R	Nrf2↑, HO-1↑, GPX4↑, ACSL4↓,COX2↓	Inhibit ferroptosis	Hu et al. (2022)
Rehmannioside A	81720-05-0	C_21_H_32_O_15_	MCAO/R, OGD/R	pPI3K↑, pAkt-1↑,Nrf2↑,HO-1↑, ROS↓, SLC7A11↓, GPX4↑	Inhibit ferroptosis	Fu et al. (2022)

### 2.4 Repairing damaged blood-brain barrier

BBB forms a mechanical and functional barrier between the systemic circulation and the central nervous system, and its integrity is important for homeostasis and function of CNS (Chen et al., 2012; Mirshekari Jahangiri et al., 2020). BBB disruption caused by CIRI contributed to vasogenic brain edema, which ultimately aggravates ischemic brain injury (Huang et al., 2018). Reperfusion after ischemia leads to progressive deterioration in BBB permeability, resulting in cytotoxic edema, ionic edema, vasogenic edema, and hemorrhagic transformation (Abdullahi et al., 2018; Khoshnam et al., 2018). Matrix metalloproteinases (MMPs) are regulators of capillary permeability, and activation of MMPs can change the integrity of BBB, thereby increasing the permeability of the BBB (Rosenberg et al., 1998; Asahi et al., 2001). Tissue inhibitors of metalloproteinases (TIMPs) are crucial regulators of BBB function, as they can inhibit MMP activity and reduce BBB permeability following CIRI (Fujimoto et al., 2008).

It has been reported that a variety of TCM monomers could repair BBB, such as various saponins (ginsenoside Rg1, astragaloside IV, etc.), a variety of phenols (sparganin C, curcumin, etc.) and other TCM monomers. Saponins could upregulate the expression of tight junctions (TJs) and downregulate the expression of MMP-9 and AQP-4, thus repaired CIRI-induced BBB disorder. In MCAO/R rats, ginsenoside-Rg1 (20 mg/kg for 14 days) inhibited the expression of MMP-9 and AQP-4, while promoted TJs to improve the permeability of BBB (Xie et al., 2015). It is also found that astragaloside IV maintained the integrity of BBB. Astragaloside IV (20 mg/kg) could reduced the expression of MMP-9 and AQP4, and at the same time increased TJs to improve the permeability of BBB, thus eased the damage in the MCAO/R rat model (Li et al., 2013). It has been reported that phenols are involved in the integrity of BBB by regulating the expression of MMPs and TIMPs. Curcumin (300 mg/kg) was found to reduce neurological scores, infarct volume, morphological changes, Evans blue leakage, and immunoglobulin G extravasation, as well as BBB injury and neutrophil infiltration. BBB could also be protected against CIRI by up-regulating tight junction protein (TJP) and decreasing the expression of AQP4 and brain water content (Wu et al., 2021b). In addition, sparganin C (30 mg/kg) upregulates TIPM1/MMP protein ratio by activating PI3K/AKT/mTOR pathway, promoting BBB integrity after injury in MCAO/R rat models (Liang et al., 2022). In addition to the above saponins and phenolic TCM, quercetin (Yang et al., 2022b), methylophiopogonanone A (Lin et al., 2015), magnolol (Liu et al., 2017), etc., could also repair CIRI induced blood-brain barrier disorders by regulating the expression levels of MMPs, TIMP and AQP4 (Table 4).

**TABLE 4:** Effects and mechanisms of different TCM monomers on repairing blood-brain barrier and neovascularization in cerebral ischemia-reperfusion injury.

TCM monomers	CAS No.	Molecular formula	Method of model	Mechanisms	•Effects	References
**Ginsenoside Rg1**	22427-39-0	C_42_H_72_O	MCAO/R	Evans Blue (EB)↑, PAR-1↓, AQP-4↓	Maintain BBB	Xie et al. (2015)
**Astragaloside IV**	84867-43-4	C_41_H_68_O_14_	MCAO/R	MMP-9↓, AQP-4↓, TJs↑	Maintain BBB	Li et al. (2013)
**Curcumin**	458–37-7	C_21_H_20_O_6_	MCAO/R	AQP-4↓, TJs↑	Maintain BBB	Wu et al. (2021b)
**Sparganin C**	5147-17-1	C_18_H_18_N_2_O_3_	MACO/R	PI3K/AKT↑, TIMP↑, MMPs↓	Maintain BBB	Liang et al. (2022)
**Quercetin**	117–39-5	C_15_H_10_O_7_	MACO/R	MMPs↓, TIMP↑, AQP4 ↓	Maintain BBB	Yang et al. (2022b)
**Methylophiopogonanone A**	74805-92-8	C_19_H_18_O_6_	MACO/R	MMPs↓, TIMP↑, AQP4 ↓	Maintain BBB	Lin et al. (2015)
**Magnolol**	528–43-8	C_18_H_18_O_2_	MACO/R	TIMP↑, MMPs↓, AQP4↓	Maintain BBB and reduce cerebral edema	Shen et al. (2022)
**Ginsenoside F1**	53963-43-2	C_36_H_62_O_9_	MACO/R	Shh↑, VEGF↑, Ang-1↑	Promote angiogenesis	Zhang et al. (2019b)
**Engeletin**	572–31-6	C_21_H_22_O_10_	MACO/R	VEGF↑, Ang-1↑, capillary density↑	Promote angiogenesis	Liu et al. (2021)
**DI-3-n-butylphthalein**	6066-49-5	C_12_H_14_O_2_	MACO/R	Nrf2↑, VEGF↑, Ang-1↑	Promote angiogenesis	Huang et al. (2021b)
**Salvianolic acid B**	121521-90-2	C_36_H_30_O_16_	MACO/R	VEGF↑, Ang-1↑	Promote angiogenesis	Bi et al. (2022)
**Morroniside**	25406-64-8	C_17_H_26_O_11_	MACO/R	VEGF↑, Ang-1↑	Promote angiogenesis	Liu et al. (2016b)

### 2.5 The role of promoting angiogenesis

A Angiogenesis is a physiological process that involves the growth of new capillaries from existing blood vessels through sprouting. It serves as a natural defense mechanism in humans and animals against ischemic injury by restoring oxygen and nutrient supply to affected tissues, ultimately promoting long-term functional recovery (Slevin et al., 2006; Beck and Plate, 2009). Angiogenesis is regulated by a delicate balance between angiogenic growth factors (such as vascular endothelial growth factor (VEGF), transforming growth factor β (TGF-β), basic fibroblast growth factor 2 (b-FGF2), platelet-derived growth factor (PDGF), etc.) and angiogenic inhibitors, which govern endothelial cell migration and proliferation. In response to CIRI, hypoxia triggers the release of angiogenic factors within the existing vascular system, resulting in elevated levels of angiogenic growth factors in microvessels and facilitating neurovascularization (Fan and Yang, 2007). Released VEGF binds to vascular endothelial growth factor receptor (VEGFR) on vascular endothelial cells, initiating capillary formation (Hayashi et al., 2003). Angiopoietin 1 (Ang1), an endogenous ligand of the endothelial-specific receptor tyrosine kinase Tie-2, plays a role in promoting vascular endothelial integrity, stability, and maintenance (Chen et al., 2009).

Various TCM monomers have been reported to alleviate CIRI by promoting angiogenesis (Shen et al., 2022). Saponins in CIRI promoted angiogenesis after CIRI by up-regulating expression of vascular growth factors (VEGF, TGF-β, b-FGF2, PDGF). *In vivo* and *in vitro* experiments, Notoginsenoside R1 restored cerebral blood flow by improving the structure of cerebral microvascular endothelial cells and up-regulating the expression of various angiogenic factors (Zhu et al., 2021). Meanwhile, ginsenoside F1 (50 mg/Kg/day for 14 days) could improve focal cerebral blood perfusion by promoting angiogenesis and increasing microvascular density, thus alleviated CIRI in rats with MCAO (Zhang et al., 2019b). The VEGF/vasohibin and Ang-1/Tie-2 signaling pathway are involved in angiogenesis and maturation. In MCAO/R rat model, engeletin upregulated the expression of vascular endothelial growth factor VEGF and Ang-1, increased capillary density and enhanced angiogenesis in the ischemic boundry zones (Liu et al., 2021) (Figure 2). In addition to saponins, other TCM monomers such as DI-3-n-butylphthalein (Huang et al., 2021b), salvianolic acid B (Bi et al., 2022), morroniside (Liu. et al., 2016b) and cardamonin (Ni et al., 2022) can also promote angiogenesis in CIRI (Table 4).

### 2.6 Inhibition of glutamate excitatory toxicity/Ca^2+^ overload

Elevated levels of glutamate can lead to the overstimulation of glutaminergic receptors on postsynaptic neurons, resulting in excitotoxicity. Glutamate binds to glutamate alpha-amino-3-hydroxy-5-methyl-4-isoxazole-propionate (AMPA) receptors and kainate (KA) receptors, causing the opening of Na + channels and an influx of Na+. This acute cell swelling and neuronal death can occur. N-methyl-D-aspartate (NMDA) receptors, when bound by a large amount of glutamate, open Ca^2+^ channels, leading to excessive Ca^2+^ influx. Intracellular mechanisms responsible for reducing Ca^2+^ levels, such as the calcium pump (PMCA pump) and exchange body (NCX exchange), become damaged due to calcium overload. In addition to glutamate excitotoxicity, other factors, such as voltage-gated calcium channels (mainly L-VGCCs) and ligand-gated calcium channel overactivation during energy deficiency, can also cause abnormal increases in intracellular Ca^2^+ concentration.

Some studies have confirmed that TCM monomer could inhibit glutamate excitatory toxicity and Ca^2^+ overload to alleviate CIRI. In both *in vitro* and *in vivo* studies, baicalin protected neurons from glutamate toxicity by protecting glutamine synthetase of astrocytes from ROS-induced carbylation and degradation of 20 S proteasome, increased glutamate-processing capacity of astrocytes (Song et al., 2020). In addition, ginsenoside Rd (50 mg/kg *in vivo*, 10 μmol *in vitro*) also reduced the excitotoxic injury of neurons by reducing NMDA receptor 2B subunit (NR2B) and its phosphorylation (Xie et al., 2016). In TCM monomers effect of Ca^2+^ overload, astragaloside IV (20 mg/kg *in vivo*, 100 μmol/L *in vitro*) alleviated CIRI by inhibiting calcium sensitive receptors and reducing apoptosis (Du et al., 2021a). In addition, another target of the regulation of calcium concentration by TCM monomers is Na + expression/Ca^2+^ exchanger (NCXs) protein. In both vitro and *in vivo* experiments have shown that intraventricular administration of cannabidiol (200 ng/rat) significantly enhanced the expression of NCX2 and NCX3 in CIRI rats, regulated calcium activity and reduced excitatory toxicity (Khaksar and Bigdeli, 2017). In addition, improving mitochondrial function could also reduce Ca^2+^ overload, for example, ligustilide mitigated Ca^2+^ overload by reducing mitochondrial division (Wu et al., 2022) (Figure 4) (Table 5).

**TABLE 5:** Effects and mechanisms of different TCM monomers on Inhibition of glutamate excitatory toxicity/Ca^2+^ overload, Mitochondrial protection in cerebral ischemia-reperfusion injury.

TCM monomers	CAS No.	Molecular formula	Method of model	Mechanisms	Effects	References
**Baicalin**	21967-41-9	C_21_H_18_O_11_	MCAO/R	Mitochondrial ROS↓, SDH↓GS↑, GS carbonylation↓, binding of 20S proteasomal to GS↓	decreases glutamate excitotoxicity	Song et al. (2020)
			OGD/R			
**Ginsenoside Rd**	52705-93-8	C_48_H_82_O_18_	MCAO/R	NR2B subunit↓, NR2B subunit phosphorylation ↓	decreases glutamate excitotoxicity	Xie et al. (2015)
			OGD/R			
**Astragaloside IV**	84687-43-4	C_41_H_68_O_14_	MCAO/R	CaSR↓, Ca2+↓, BCL2/BAX↓, AIF↑	Inhibit Ca^2+^ overload and apotosis	Du et al. (2021a)
			OGD/R			
**Cannabidiol**	13956-29-1	C_21_H_30_O_2_	MCAO/R	NCX23↑, NCX3↑	Anti-excitotoxicity and calcium regulation	Khaksar and Bigdeli (2017)
**Polydatin**	65914-17-2	C_20_H_22_O_8_	MCAO/R	MMP↓Bcl-2↑Bax ↓	Maintain Mitochondrial dysfuction and Anti-apoptosis	Gao et al. (2016)
				Caspase3↓, caspase9↓		
**Curcumin**	458–37-7	C_21_H_20_O_6_	MCAO/R	SIRT1↑, Ac-p53↓, MMP↓, Bax↓, Bcl-2↑, ATP↑LC3-II/LC3-I↑	Maintain Mitochondrial dysfuction and Anti-apoptosis Promote mitophagy	Miao et al. (2016)
			OGD/R			
**Chikusetsu saponin V**	51415-02-2	C_42_H_66_O_14_	MCAO/R	p-AMPK↑, SIRT-1↑, Ac- PGC-1α↑, ROS↓, mitochondrial respiratory↑	Maintain Mitochondrial dysfuction and anti-oxidative stress	Wang and Xu (2020)
			OGD/R			
**Ligustilide**	81944-09-4	C_12_H_14_O_2_	MCAO/R	AMPK↑, Fis1↑, LC3-II/LC3-I↑, p62↓	induce mitochondrial fission and mitophagy	Zhang et al. (2021b)
			OGD/R	PINK1↑, Parkin↑		
**β-Caryophyllene**	87–44-5	C15H24	MCAO/R	LC3-II/LC3-I↑, p62↓	induce mitophagy	Mao et al. (2022), Wu et al. (2022)
			OGD/R	PINK1↑, Parkin2↑		
**Atractylenolide III**	73030-71-4	C_15_H_20_O_3_	MCAO/R	pJAK↓, 2pSTAT3↓, Drp1↓, pDrp1↓	inhibit mitochondrial fission and anti-imflamation	Rao et al. (2022)
			OGD/R			

### 2.7 Mitochondrial protection

The excessive production of reactive oxygen species (ROS) and Ca2+ during CIRI leads to the overopening of mitochondrial permeability transition pores (MPTP), alterations in mitochondrial permeability, and disruption of membrane structure. This results in a decrease in mitochondrial membrane potential, the disappearance of ion gradients across the inner mitochondrial membrane, and ultimately mitochondrial dysfunction. In turn, mitochondrial dysfunction exacerbates energy supply disturbances, oxidative stress damage, and mitochondrial Ca^2+^ overload in brain cells. Additionally, mitochondrial dysfunction can trigger mitochondrial fission, fusion, and mitophagy, which help maintain cellular homeostasis by eliminating damaged or depolarized mitochondria.

Many studies have shown that TCM monomers could improve CIRI by improving mitochondrial function and structural integrity. A variety of TCM monomers protected mitochondrial function by improving mitochondrial membrane potential, such as polydatin and curcumin. Polydatin (30 mg/kg) improved mitochondrial membrane potential (MMP) and reduced neuronal apoptosis (Gao et al., 2016). Curcumin (350 mg/kg) increased MMP, mitochondrial complex I activity and mitochondrial cytochrome enzyme C levels to alleviate mitochondrial dysfunction and thus reduced cell apoptosis (Miao et al., 2016). Another study found that low-dose curcumin (100 mg/kg *in vivo*, 5 µM *in vitro*) could improve mitochondrial function, increase LC3 II, mitochondrial marker VDAC1 colocalization, LC3-II/LC3-I ratio, and enhance mitophagy (Wang and Xu, 2020). Chikusetsu saponin V (50 mg/kg *in vivo*, 50 μM *in vitro*) reduced deacetylation of peroxisome proliferator-activated receptor γ coactivator-1α (PGC-1α) through AMPK/SIRT-1 pathway, downregulated ROS and maintained mitochondrial respiration (Zhang et al., 2021b). In addition, ligustilide (20 mg/kg *in vivo*, 20 μM *in vitro*) promoted DRP1-mediated mitochondrial fission and induced mitophagy by activating AMPK, thereby alleviating CIRI (Khaksar and Bigdeli, 2017). It is also found that ligustilide (20 mg/kg *in vivo*, 20 μM *in vitro*) could improve CIRI by enhancing PINK1/Parkin-dependent mitophagy. Different TCM monomers with the same mechanism of action include Caryophyllene (Mao et al., 2022; Rao et al., 2022). In addition, atractylenolide III (10 mg/kg *in vivo*, 1 μM *in vitro*) from *Atractylodes macrocephala* Koidz could also alleviate neuroinflammation by reducing microglial Drp1 translocation and phosphorylated mitochondrial division via JAK2/STAT3 (Zhou et al., 2019) (Figure 4) (Table 5).

### 2.8 Others

Apart from the aforementioned effects, TCM monomers also play a significant role in neurotransmitter release, neurogenesis, microglia polarization, neurovascular unit function, and related complications such as cognitive impairment and depression in the context of CIRI. In an MCAO model, salidroside (80 mg/kg) could enhance the regulation of tyrosine hydroxylase (TH) in the striatum and SNpc to increase the content of dopamine (DA), homovanillic acid (HVA) and 3, 4-dihydroxyphenylacetic acid (DOPAC) in the striatum. Thus, behavioral disorders caused by CIRI improved (Zhong et al., 2019). Astragaloside IV (2 μg/kg) promoted the differentiation of neural stem cells and increased neurogenesis (Ni et al., 2020). In addition, the polarization of microglia is another target of TCM monomers. Baicalein (100 mg/kg) reduced the polarization of microglia to M1 type and inhibited neuroinflammation (Ran et al., 2021b). Neurovascular unit dysfunction is an important pathological process during CIRI. In recent years, it has been found that TCM monomers could improve neurovascular unit dysfunction. It has been reported that 270 mg/kg dose of morroniside in MCAO model significantly increased the recruitment of endothelial progenitor cells (EPCs), the expression of angiogenic factors and the formation of new blood vessels around infarction, thereby protecting the integrity of neurovascular unit microvessels and improving cerebral ischemia reperfusion injury (Sun et al., 2014). In addition, studies have also found that TCM monomers improved CIRI complications such as cognitive impairment and depression symptoms. Curcumin (300 mg/kg, 20 μmol/L) has been reported to alleviate neuroinflammation, oxidative stress and neuronal apoptosis by inhibiting the expression of miR-7-5p/RelA p65, improving cognitive dysfunction after CIRI (Xu et al., 2019). Hydroxysafflor Yellow A (16 mg/kg) improved cognitive impairment by rescuing damaged long-term enhancement (LTP) in the hippocampus of MCAO/R rats (Yu et al., 2018). In addition to improving neurovascular unit dysfunction and cognitive impairment, TCM monomers played an important role in alleviating post-stroke depression. Astragaloside VI (2 μg/kg, 100 nM) miligated post-stroke depression by increasing DA and 5-HT release through upregulation of the MEK/ERK pathway mediated by the neurotrophic factor neuregulin 1 (NRG-1) (Chen et al., 2022b). Curcumin (100 mg/kg *in vivo*) inhibited the activation of calcium channels by inhibiting P2X7 receptor (P2X7R) to reduce the symptoms of post-stroke depression in rats (Wang et al., 2020b). In addition, some scholars have found that the ginsenoside Rb1 could improve lung and intestinal barrier damage after cerebral ischemia-reperfusion (Su et al., 2022). Butylphthalide a traditional Chinese medicine monomer that has been used in clinical practice, could increase blood perfusion by improving vasoconstriction and reduce thrombosis at the dose of 90 mg/Kg (Qin et al., 2019).

To conclude, TCM monomers can effectively prevent and treat cerebral ischemia-reperfusion injury by employing various mechanisms such as inhibiting oxidative stress, inflammation, glutamate excitatory toxicity, Ca^2^+ overload and programmed cell death. Additionally, they facilitate the repair of the blood-brain barrier, promote angiogenesis, improve neurotransmitter release, protect neurovascular unit integrity, and alleviate post-stroke cognitive depression and systemic symptoms. Therefore, it is considered that the effect of TCM monomers on ischemia-reperfusion injury is multi-target, and the scope of research has also expanded from the initial neurons to other cellular components such as microglia, astrocytes and neurovascular units.

## 3 Traditional Chinese medicine monomers related to cerebral ischemia-reperfusion injury

Due to the intricate pharmacology of TCM preparations, pinpointing their exact mechanism of action can be challenging. However, TCM monomers offer distinct advantages in terms of their well-defined mechanisms, predictable pharmacological actions, and reduced potential for drug interactions. As a result, the use of TCM monomers in the treatment of various diseases, including CNS injury conditions like CIRI caused by diverse mechanisms, has garnered significant attention in recent years.

### 3.1 Phenols

**Salvianolic acid B** Salvianolic acid B is a water-soluble product of *Salvia miltiorrhiza* Bunge, which can be used to treat cardio-cerebrovascular diseases due to its antioxidant and anti-inflammatory properties (Zhou et al., 2005). In stroke, salvianolic acid B could reduce brain injury by inhibiting cell apoptosis and inflammation, reducing brain edema, and increasing neurological function score (Lv et al., 2015).

**Curcumin** Curcumin is a plant component isolated from *Curcuma longa* L with anti-inflammatory, antibacterial, anti-fibrosis, and antioxidant effects, and has multiple effects on the central nervous system (Priyadarsini, 2014). Curcumin attenuated autophagy activity by mediating Phosphoinositide 3-Kinase (PI3K)/Protein Kinase B (Akt)/the mammalian target of rapamycin (mTOR) pathway, inhibited inflammation by regulating Toll-like receptor 4 (TLR4)/p38/mitogen activated protein kinase (MAPK) pathway (Huang et al., 2018), and protected BBB from destruction by inducing oxidative stress response, leukocyte infiltration, complement activation and mitochondrial biogenesis disorder. It played a protective role in CIRI (Bavarsad et al., 2019).

### 3.2 Glycosides

**Astragaloside IV** Astragaloside IV is an effective component of *Astragalus mongholicus* Bunge, which is widely used in the prevention and treatment of cardiovascular and cerebrovascular diseases in China (Zhang et al., 2020d). Many studies have confirmed that astragaloside IV could improve neurological deficits, reduce infarct volume and BBB permeability, and provide neuroprotection during CIRI through its anti-inflammatory, anti-apoptotic and anti-oxidative effects (Wang et al., 2017; Zhang et al., 2019a; Xu et al., 2020).

**Ginsenoside Rg1** Ginsenoside Rg1 is an active component isolated from the total saponins of *Panax quinquefolius* L. Rg1 could significantly reduce the infarct volume and reduce the neurological deficit caused by cerebral ischemia/reperfusion (Zeng et al., 2014). The reasons may be related to anti-oxidative stress, anti-inflammatory and anti-apoptotic effects as well as promotion of BBB repair and prevention of calcium overload (Yang et al., 2020).

### 3.3 Terpenoids

**Ginkgolide B** Ginkgolide B (GB) is a terpenolactone component of *Ginkgo biloba* L Extract, which has neuroprotective and antioxidant effects (Singh et al., 2019). GB has been shown to inhibit I/R-induced nuclear factor NF-kappaB (NF-κB) and microglia activation and pro-inflammatory cytokine production in focal I/R models, and significantly reduce infarct volume, brain edema, and neurological deficits (Gu et al., 2012).

**Artemisinin** Artemisinin is a sesquiterpene lactone peroxide extracted from *Artemisia annua* L leaves (Mohammadi et al., 2020). In ischemic stroke, artemisinin can significantly inhibit apoptosis, oxidative stress and neuroinflammation (Peng et al., 2022). Artesunate is a semi-synthetic antimalarial compound derived from artemisinin (Morris et al., 2011). Artesunate may be a potential therapeutic agent for ischemic cerebrovascular disease (Zhang et al., 2020e). Studies have shown that artesunate ameliorated damage caused by TBI via its anti-inflammatory activity and regulated neurotrophic factors that play a key role in neuronal survival (Gugliandolo et al., 2018). Artesunate also protected BBB by activating sphingosine-1-phosphate receptor-1 (S1P1), enhancing PI3K activation and stabilizing beta-catenin in SAH mice (Zuo et al., 2017).

### 3.4 Flavonoids

**Icariin** Icariin is the main component of flavonoids extracted from *Epimedium sagittatum*, which has been proven to have potential preventive and therapeutic effects on nervous system diseases. Because of its biological activities related to anti-apoptosis, anti-oxidation and anti-inflammatory effects, it could reduce the expression of inflammatory factors and accelerate the recovery of motor function after nerve injury (Jia et al., 2019).

**Puerarin** Puerarin is an isoflavone compound extracted from *Neorautanenia mitis*, which can protect organs from I/R injury through various mechanisms, such as reducing lactic acid production, inhibiting inflammatory response, antioxidant, promoting angiogenesis and inhibiting autophagic reaction, etc (Gao et al., 2022). Puerarin improved the outcome of CIRI, reduce infarct volume and improve nerve function. Puerarin could remove free radicals, increase cerebral blood flow, play a neuroprotective and anti-inflammatory role in CIRI, reduce infarct volume, and thus improve the outcome of CIRI (Zhou et al., 2014).

**Vitexin** Vitexin is a bioactive flavonoid compound from *Ficus thonningii* Blume with antioxidant, anti-inflammatory, antibacterial, neuroprotective and cardioprotective biological activities (Hu et al., 2023). In current studies on CIRI, vitexin has been found to have protective properties on CIRI by regulating MAPK and B-lymphocyte tumor-2 (Bcl-2)/Bcl-2 related x protein (Bax) signaling pathway (Jiang et al., 2018). MCAO-induced autophagy can also be inhibited by activating mTOR/the mammalian autophagy-initiating kinase Ulk1 pathway to reduce oxidative stress damage and inflammation (Hongyun et al., 2017). In addition, it also played a protective role in brain injury caused by CIRI through regulating brain endothelial permeability (Cui et al., 2019).

**Baicalin** Baicalin is the main component isolated from the dried root of *Scutellaria baicalensis* Georgi, which has antioxidant, anti-inflammatory and many other bioactive properties and has been used in the treatment of various diseases (Jiang et al., 2020; Nam et al., 2020). Previous studies have confirmed that baicalin can participate in multi-stage cascade reaction after ischemic stroke and alleviate focal cerebral ischemia/reperfusion injury (Long et al., 2022).

### 3.5 Others

**Ligustrazine** Ligustrazine is the main active ingredient of *Conioselinum anthriscoides ‘Chuanxiong’*, which is widely used in the treatment of ischemic cerebrovascular disease due to its function of promoting blood circulation and migration, differentiation, and proliferation of neural stem cells (Guo et al., 1983). Ligustrazine is favored in the treatment of ischemic cerebrovascular diseases, which could protect neurons in a variety of ways (Du et al., 2021b). In focal ischemic stroke, ligustrazine has protective effects such as lowering the blood-brain barrier, dilating cerebral vessels, preventing thrombosis, anti-inflammatory, antioxidant, and activating microglia cells (Lin et al., 2021).

**Phillyrin** Phillyrin is an important active ingredient extracted from *Forsythia suspensa* (Thunb.) Vahl, which has anti-inflammatory, antioxidant, and other physiological functions (Han et al., 2018), and could reduce I/R damage by inhibiting neuronal apoptosis and autophagy pathways (Chen et al., 2022a).

**Berberine** Berberine (BBR) is the major constitutes of *Coptis chinensis* Franch. Because BBR can cross the blood-brain barrier, BBR can play a neuroprotective role in ischemic brain injury (Liu et al., 2019). In CIRI, BBR may reduce neuronal apoptosis by reducing the expression of caspase-3 and caspase-9 and increasing the proportion of Bcl-2/Bax (Sun et al., 2020). In addition, BBR reduced ischemic brain injury by decreasing the level of intracellular reactive oxygen species (ROS) and inhibiting mitochondrial apoptosis pathway (Zhou et al., 2008).

**Dl-3-N-butylphthalide (NBP)**:DI-3-N-Butylphthalide (NBP) is a family of compounds initially isolated from the seeds of *Apium graveolens* L. It has shown significant neuroprotective effects in cerebral ischemic-reperfusion injury. NBP has peotective effects such as inhibiting platelet aggregation, preventing vasoconstriction, reducing mitochondrial damage, down-regulating cell apoptosis, attenuating oxidative stress and promoting neurogenesis (Abdoulaye and Guo, 2016).

## 4 Discussion and future perspectives

### 4.1 Limitations of current research on traditional Chinese medicine monomers

The current research on TCM monomers and their protective effects in CIRI is primarily based on preclinical experimental studies (Xie et al., 2018; Li et al., 2022c). However, there are certain limitations in the epidemiological studies conducted on TCM monomers and CIRI. These limitations include study design, small sample sizes, variations in the dosage of TCM monomers, and differences in the administration modes. Most of the studies in the field of CIRI and TCM monomers are still at the cellular or rodent stage. Although some TCM monomers have progressed to the preclinical research stage, the route of administration plays a significant role in determining the therapeutic efficacy and safety of the drug (Fleischmann et al., 2019). Oral administration, for instance, is subject to the first-pass effect, resulting in only a small portion of the active ingredient crossing the blood-brain barrier to reach the intended site. Moreover, oral administration may also induce various adverse reactions, thereby limiting its clinical utility (He et al., 2017). The specific mechanism and optimal dosage of TCM monomers for treating CIRI remain unclear. Additionally, The current research on TCM monomers in the treatment of CIRI lacks large-scale, multi-center clinical trials, making it challenging to determine the exact effective dose, optimal dose, safety profile (including potential side effects), and feasibility of these monomers.

### 4.2 Future research trends of traditional Chinese medicine monomers

Future studies should focus on conducting large-scale clinical trials and comprehensive pharmacological investigations to further elucidate the specific mechanisms by which TCM monomers exert their effects in CIRI. These studies should aim to provide robust medical evidence that can guide the development of clinical treatments for patients with CIRI. Furthermore, it is crucial to explore more suitable drug delivery systems, such as nanoparticles or hydrogels, to facilitate the passage of drugs across the blood-brain barrier. This would enhance the retention time, bioavailability, and concentration of drugs at the site of injury, while reducing the frequency of administration, side effects, and toxicity. By doing so, the therapeutic efficacy can be improved, and better biosafety can be ensured *in vivo* (Li et al., 2014; Thakur et al., 2018).

## 5 Conclusion

Cerebral ischemia-reperfusion injury is an extremely destructive process in the brain with devastating effects on patients. The rapid emergence of various pathological processes makes functional recovery of damaged brain tissue challenging and often leads to severe functional deficits. The mechanisms underlying this extensive damage involve increased oxidative stress, disruption of the blood-brain barrier, inflammation, and programmed cell death. Consequently, effective treatment options for rebuilding severely damaged neurological function in CIRI patients are currently lacking. In order to address this challenge, research efforts are being directed towards tissue engineering, cell transplantation and molecule-target therapies are being applied to the field of CIRI. This review explicitly describes the molecular mechanism of TCM monomers during CIRI in recent years. The intervention of TCM monomers in CIRI is characterized by a multi-target approach, going beyond the previous emphasis solely on neuronal neuroprotection. The protective effect of TCM monomers on CIRI involves almost all cellular components of the nervous system, in the protection of neurovascular units, while taking into account supporting structures such as astrocytes, microglia and blood-brain barrier. Moreover, the influence of TCM monomers extends beyond the injury itself and encompasses post-stroke depression and anxiety. Furthermore, new mechanisms of action for TCM monomers have been discovered, expanding beyond the previously recognized pathways such as anti-inflammation via the NF-κB pathway and antioxidation through the Nrf pathway to include emerging pathways such as pyroptosis and ferroptosis. However, CIRI is a complex and multifactorial process, and despite the successful clinical application of certain TCM monomers such as butylphthalide and ligustrazine, many traditional Chinese medicine monomers remain in the preclinical trial stage. Before transitioning to clinical practice, it is crucial to address several unresolved issues. Comprehensive preclinical pharmacological and toxicological tests, as well as clinical trials, are necessary to evaluate the effectiveness and safety of TCM monomers. Furthermore, the potential for unpredictable side effects associated with certain Chinese medicine monomers necessitates careful consideration of their use.

In conclusion, taking a holistic approach, it is important to emphasize and extensively explore TCM monomers as a basis for anti-CIRI treatment. This approach has the potential to reduce the incidence and mortality of CIRI and prolong the survival of patients.

## References

[B1] Abdoulaye I. A. Guo Y. J. (2016). A review of recent advances in neuroprotective potential of 3-N-butylphthalide and its derivatives. Biomed. Res. Int. 2016, 5012341. 10.1155/2016/5012341 28053983 PMC5178327

[B2] Abdullahi W. Tripathi D. Ronaldson P. T. (2018). Blood-brain barrier dysfunction in ischemic stroke: targeting tight junctions and transporters for vascular protection. Am. J. Physiol. Cell. Physiol. 315 (3), C343–c356. 10.1152/ajpcell.00095.2018 29949404 PMC6171039

[B3] An P. Xie J. Qiu S. Liu Y. Wang J. Xiu X. (2019). Hispidulin exhibits neuroprotective activities against cerebral ischemia reperfusion injury through suppressing NLRP3-mediated pyroptosis. Life Sci. 232, 116599. 10.1016/j.lfs.2019.116599 31247210

[B4] Anrather J. Iadecola C. (2016). Inflammation and stroke: an overview. Neurotherapeutics 13 (4), 661–670. 10.1007/s13311-016-0483-x 27730544 PMC5081118

[B5] Arvin B. Neville L. F. Barone F. C. Feuerstein G. Z. (1996). The role of inflammation and cytokines in brain injury. Neurosci. Biobehav Rev. 20 (3), 445–452. 10.1016/0149-7634(95)00026-7 8880734

[B6] Asahi M. Wang X. Mori T. Sumii T. Jung J. C. Moskowitz M. A. (2001). Effects of matrix metalloproteinase-9 gene knock-out on the proteolysis of blood-brain barrier and white matter components after cerebral ischemia. J. Neurosci. 21 (19), 7724–7732. 10.1523/JNEUROSCI.21-19-07724.2001 11567062 PMC6762894

[B7] Bavarsad K. Barreto G. E. Hadjzadeh M. A. R. Sahebkar A. (2019). Protective effects of curcumin against ischemia-reperfusion injury in the nervous system. Mol. Neurobiol. 56 (2), 1391–1404. 10.1007/s12035-018-1169-7 29948942

[B8] Beck H. Plate K. H. (2009). Angiogenesis after cerebral ischemia. Acta Neuropathol. 117 (5), 481–496. 10.1007/s00401-009-0483-6 19142647

[B9] Bi S. J. Dong X. Y. Wang Z. Y. Fu S. J. Wang Z. Y. (2022). Salvianolic acid B alleviates neurological injury by upregulating stanniocalcin 1 expression. Ann. Transl. Med. 10 (13), 739. 10.21037/atm-21-4779 35957712 PMC9358494

[B10] Broughton B. R. Reutens D. C. Sobey C. G. (2009). Apoptotic mechanisms after cerebral ischemia. Stroke 40 (5), e331–e339. 10.1161/STROKEAHA.108.531632 19182083

[B11] Chen C. H. Hsieh C. L. (2020). Effect of acupuncture on oxidative stress induced by cerebral ischemia-reperfusion injury. Antioxidants (Basel) 9 (3), 248. 10.3390/antiox9030248 32204376 PMC7139408

[B12] Chen G. H. Li X. L. Deng Y. Q. Zhou F. M. Zou W. Q. Jiang W. X. (2019). The molecular mechanism of EPO regulates the angiogenesis after cerebral ischemia through AMPK-KLF2 signaling pathway. Crit. Rev. Eukaryot. Gene Expr. 29 (2), 105–112. 10.1615/CritRevEukaryotGeneExpr.2019029018 31679265

[B13] Chen J. Cui X. Zacharek A. Chopp M. (2009). Increasing Ang1/Tie2 expression by simvastatin treatment induces vascular stabilization and neuroblast migration after stroke. J. Cell. Mol. Med. 13 (7), 1348–1357. 10.1111/j.1582-4934.2008.00380.x 18544044 PMC3710660

[B14] Chen S. Zhang S. Wu H. Zhang D. You G. You J. (2022a). Protective effect of phillyrin against cerebral ischemia/reperfusion injury in rats and oxidative stress-induced cell apoptosis and autophagy in neurons. Bioengineered 13 (3), 7940–7950. 10.1080/21655979.2022.2042142 35291908 PMC9278963

[B15] Chen W. Guo Y. Yang W. Zheng P. Zeng J. Tong W. (2015). Protective effect of ginsenoside Rb1 on integrity of blood-brain barrier following cerebral ischemia. Exp. Brain Res. 233 (10), 2823–2831. 10.1007/s00221-015-4352-3 26070903

[B16] Chen X. Shen J. Zhou Q. Jin X. Liu H. Gao R. (2022b). Astragaloside VI ameliorates post-stroke depression via upregulating the NRG-1-mediated MEK/ERK pathway. Pharm. (Basel) 15 (12), 1551. 10.3390/ph15121551 PMC9784132 36559001

[B17] Chen X. Threlkeld S. W. Cummings E. E. Juan I. Makeyev O. Besio W. G. (2012). Ischemia-reperfusion impairs blood-brain barrier function and alters tight junction protein expression in the ovine fetus. Neuroscience 226, 89–100. 10.1016/j.neuroscience.2012.08.043 22986172 PMC3490041

[B18] Chen Y. Zhang L. Gong X. Gong H. Cheng R. Qiu F. (2020). Iridoid glycosides from Radix Scrophulariae attenuates focal cerebral ischemia-reperfusion injury via inhibiting endoplasmic reticulum stress-mediated neuronal apoptosis in rats. Mol. Med. Rep. 21 (1), 131–140. 10.3892/mmr.2019.10833 31746404 PMC6896402

[B19] Cho J. J. Xu Z. Parthasarathy U. Drashansky T. T. Helm E. Y. Zuniga A. N. (2019). Hectd3 promotes pathogenic Th17 lineage through Stat3 activation and Malt1 signaling in neuroinflammation. Nat. Commun. 10 (1), 701. 10.1038/s41467-019-08605-3 30741923 PMC6370850

[B20] Clausen B. H. Lambertsen K. L. Dagnæs-Hansen F. Babcock A. A. von Linstow C. U. Meldgaard M. (2016). Cell therapy centered on IL-1Ra is neuroprotective in experimental stroke. Acta Neuropathol. 131 (5), 775–791. 10.1007/s00401-016-1541-5 26860727 PMC4835531

[B21] Cui Y. H. Zhang X. Q. Wang N. D. Zheng M. D. Yan J. (2019). Vitexin protects against ischemia/reperfusion-induced brain endothelial permeability. Eur. J. Pharmacol. 853, 210–219. 10.1016/j.ejphar.2019.03.015 30876978

[B22] Datta A. Sarmah D. Mounica L. Kaur H. Kesharwani R. Verma G. (2020). Cell death pathways in ischemic stroke and targeted pharmacotherapy. Transl. Stroke Res. 11 (6), 1185–1202. 10.1007/s12975-020-00806-z 32219729

[B23] de Oliveira M. R. de Souza I. C. C. Brasil F. B. (2021). Mitochondrial protection and anti-inflammatory effects induced by emodin in the human neuroblastoma SH-SY5Y cells exposed to hydrogen peroxide: involvement of the AMPK/Nrf2 signaling pathway. Neurochem. Res. 46 (3), 482–493. 10.1007/s11064-020-03181-1 33219897

[B24] Derex L. Cho T. H. (2017). Mechanical thrombectomy in acute ischemic stroke. Rev. Neurol. Paris. 173 (3), 106–113. 10.1016/j.neurol.2016.06.008 28238346

[B25] Dong J. Zhang X. Wang S. Xu C. Gao M. Liu S. (2020). Thymoquinone prevents dopaminergic neurodegeneration by attenuating oxidative stress via the Nrf2/ARE pathway. Front. Pharmacol. 11, 615598. 10.3389/fphar.2020.615598 33519481 PMC7840486

[B26] Dorweiler B. Pruefer D. Andrasi T. B. Maksan S. M. Schmiedt W. Neufang A. (2007). Ischemia-reperfusion injury: pathophysiology and clinical implications. Eur. J. Trauma Emerg. Surg. 33 (6), 600–612. 10.1007/s00068-007-7152-z 26815087

[B27] Du H. Y. Wang R. Li J. L. Luo H. Xie X. Y. Yan R. (2021b). Ligustrazine induces viability, suppresses apoptosis and autophagy of retinal ganglion cells with ischemia/reperfusion injury through the PI3K/Akt/mTOR signaling pathway. Bioengineered 12 (1), 507–515. 10.1080/21655979.2021.1880060 33522374 PMC8806313

[B28] Du S. J. Zhang Y. Zhao Y. M. Dong Y. J. Tang J. L. Zhou X. H. (2021a). Astragaloside IV attenuates cerebral ischemia-reperfusion injury in rats through the inhibition of calcium-sensing receptor-mediated apoptosis. Int. J. Mol. Med. 47 (1), 302–314. 10.3892/ijmm.2020.4777 33416112 PMC7723498

[B29] Eefting F. Rensing B. Wigman J. Pannekoek W. J. Liu W. M. Cramer M. J. (2004). Role of apoptosis in reperfusion injury. Cardiovasc Res. 61 (3), 414–426. 10.1016/j.cardiores.2003.12.023 14962473

[B30] Fan X. Elkin K. Shi Y. Zhang Z. Cheng Y. Gu J. (2020). Schisandrin B improves cerebral ischemia and reduces reperfusion injury in rats through TLR4/NF-κB signaling pathway inhibition. Neurol. Res. 42 (8), 693–702. 10.1080/01616412.2020.1782079 32657248

[B31] Fan Y. Yang G. Y. (2007). Therapeutic angiogenesis for brain ischemia: a brief review. J. Neuroimmune Pharmacol. 2 (3), 284–289. 10.1007/s11481-007-9073-3 18040863

[B32] Fleischmann R. M. Bliddal H. Blanco F. J. Schnitzer T. J. Peterfy C. Chen S. (2019). A phase II trial of lutikizumab, an anti-interleukin-1α/β dual variable domain immunoglobulin, in knee osteoarthritis patients with synovitis. Arthritis Rheumatol. 71 (7), 1056–1069. 10.1002/art.40840 30653843

[B33] Fu C. Wu Y. Liu S. Luo C. Lu Y. Liu M. (2022). Rehmannioside A improves cognitive impairment and alleviates ferroptosis via activating PI3K/AKT/Nrf2 and SLC7A11/GPX4 signaling pathway after ischemia. J. Ethnopharmacol. 289, 115021. 10.1016/j.jep.2022.115021 35091012

[B34] Fujimoto M. Takagi Y. Aoki T. Hayase M. Marumo T. Gomi M. (2008). Tissue inhibitor of metalloproteinases protect blood-brain barrier disruption in focal cerebral ischemia. J. Cereb. Blood Flow. Metab. 28 (10), 1674–1685. 10.1038/jcbfm.2008.59 18560439

[B35] Gao H. J. Liu P. F. Li P. W. Huang Z. Y. Yu F. B. Lei T. (2015). Ligustrazine monomer against cerebral ischemia/reperfusion injury. Neural Regen. Res. 10 (5), 832–840. 10.4103/1673-5374.156991 26109963 PMC4468780

[B36] Gao M. Zhang Z. Lai K. Deng Y. Zhao C. Lu Z. (2022). Puerarin: a protective drug against ischemia-reperfusion injury. Front. Pharmacol. 13, 927611. 10.3389/fphar.2022.927611 36091830 PMC9449408

[B37] Gao Y. Chen T. Lei X. Li Y. Dai X. Cao Y. (2016). Neuroprotective effects of polydatin against mitochondrial-dependent apoptosis in the rat cerebral cortex following ischemia/reperfusion injury. Mol. Med. Rep. 14 (6), 5481–5488. 10.3892/mmr.2016.5936 27840959 PMC5355690

[B38] Ghandadi M. Sahebkar A. (2017). Curcumin: an effective inhibitor of interleukin-6. Curr. Pharm. Des. 23 (6), 921–931. 10.2174/1381612822666161006151605 27719643

[B39] Gu J. H. Ge J. B. Li M. Wu F. Zhang W. Qin Z. H. (2012). Inhibition of NF-κB activation is associated with anti-inflammatory and anti-apoptotic effects of Ginkgolide B in a mouse model of cerebral ischemia/reperfusion injury. Eur. J. Pharm. Sci. 47 (4), 652–660. 10.1016/j.ejps.2012.07.016 22850444

[B40] Gugliandolo E. D'Amico R. Cordaro M. Fusco R. Siracusa R. Crupi R. (2018). Neuroprotective effect of artesunate in experimental model of traumatic brain injury. Front. Neurol. 9, 590. 10.3389/fneur.2018.00590 30108544 PMC6079305

[B41] Guo H. Zhu L. Tang P. Chen D. Li Y. Li J. (2021). Carthamin yellow improves cerebral ischemia-reperfusion injury by attenuating inflammation and ferroptosis in rats. Int. J. Mol. Med. 47 (4), 52. 10.3892/ijmm.2021.4885 33576458 PMC7895518

[B42] Guo S. K. Chen K. J. Qian Z. H. Weng W. L. Qian M. Y. (1983). Tetramethylpyrazine in the treatment of cardiovascular and cerebrovascular diseases. Planta Med. 47 (2), 89. 10.1055/s-2007-969959 6844454

[B43] Han B. Jiang P. Liu W. Xu H. Li Y. Li Z. (2018). Role of daucosterol linoleate on breast cancer: studies on apoptosis and metastasis. J. Agric. Food Chem. 66 (24), 6031–6041. 10.1021/acs.jafc.8b01387 29878766

[B44] Hao M. Li X. Feng J. Pan N. (2015). Triptolide protects against ischemic stroke in rats. Inflammation 38 (4), 1617–1623. 10.1007/s10753-015-0137-x 25687641

[B45] Hasanzadeh S. Read M. I. Bland A. R. Majeed M. Jamialahmadi T. Sahebkar A. (2020). Curcumin: an inflammasome silencer. Pharmacol. Res. 159, 104921. 10.1016/j.phrs.2020.104921 32464325

[B46] Hayashi T. Noshita N. Sugawara T. Chan P. H. (2003). Temporal profile of angiogenesis and expression of related genes in the brain after ischemia. J. Cereb. Blood Flow. Metab. 23 (2), 166–180. 10.1097/01.WCB.0000041283.53351.CB 12571448

[B47] He J. Zhou D. Yan B. (2020). Eriocitrin alleviates oxidative stress and inflammatory response in cerebral ischemia reperfusion rats by regulating phosphorylation levels of Nrf2/NQO-1/HO-1/NF-κB p65 proteins. Ann. Transl. Med. 8 (12), 757. 10.21037/atm-20-4258 32647682 PMC7333167

[B48] He Z. Wang B. Hu C. Zhao J. (2017). An overview of hydrogel-based intra-articular drug delivery for the treatment of osteoarthritis. Colloids Surf. B Biointerfaces 154, 33–39. 10.1016/j.colsurfb.2017.03.003 28288340

[B49] Hongyun H. Tao G. Pengyue Z. Liqiang Y. Yihao D. (2017). Puerarin provides a neuroprotection against transient cerebral ischemia by attenuating autophagy at the ischemic penumbra in neurons but not in astrocytes. Neurosci. Lett. 643, 45–51. 10.1016/j.neulet.2017.02.009 28192195

[B50] Hu Q. Zuo T. Deng L. Chen S. Yu W. Liu S. (2022). β-Caryophyllene suppresses ferroptosis induced by cerebral ischemia reperfusion via activation of the NRF2/HO-1 signaling pathway in MCAO/R rats. Phytomedicine 102, 154112. 10.1016/j.phymed.2022.154112 35550220

[B51] Hu Z. Y. Yang Z. B. Zhang R. Luo X. J. Peng J. (2023). The protective effect of vitexin compound B-1 on rat cerebral I/R injury through a mechanism involving modulation of miR-92b/NOX4 pathway. CNS Neurol. Disord. Drug Targets 22 (1), 137–147. 10.2174/1871527321666220324115848 35331124

[B52] Huang D. Zhou J. Li W. Zhang L. Wang X. Liu Q. (2021a). Casticin protected against neuronal injury and inhibited the TLR4/NF-κB pathway after middle cerebral artery occlusion in rats. Pharmacol. Res. Perspect. 9 (2), e00752. 10.1002/prp2.752 33704926 PMC7948701

[B53] Huang L. Chen C. Zhang X. Li X. Chen Z. Yang C. (2018). Neuroprotective effect of curcumin against cerebral ischemia-reperfusion via mediating autophagy and inflammation. J. Mol. Neurosci. 64 (1), 129–139. 10.1007/s12031-017-1006-x 29243061

[B54] Huang Y. Pan L. Wu T. (2021b). Improvement of cerebral ischemia-reperfusion injury by L-3-n-butylphthalide through promoting angiogenesis. Exp. Brain Res. 239 (1), 341–350. 10.1007/s00221-020-05978-6 33180154

[B55] Iadecola C. Anrather J. (2011). The immunology of stroke: from mechanisms to translation. Nat. Med. 17 (7), 796–808. 10.1038/nm.2399 21738161 PMC3137275

[B56] Jia G. Zhang Y. Li W. Dai H. (2019). Neuroprotective role of icariin in experimental spinal cord injury via its antioxidant, anti-neuroinflammatory and anti-apoptotic properties. Mol. Med. Rep. 20 (4), 3433–3439. 10.3892/mmr.2019.10537 31432160

[B57] Jia Y. Tong Y. Min L. Li Y. Cheng Y. (2021). Protective effects of oridonin against cerebral ischemia/reperfusion injury by inhibiting the NLRP3 inflammasome activation. Tissue Cell. 71, 101514. 10.1016/j.tice.2021.101514 33676236

[B58] Jiang J. Dai J. Cui H. (2018). Vitexin reverses the autophagy dysfunction to attenuate MCAO-induced cerebral ischemic stroke via mTOR/Ulk1 pathway. Biomed. Pharmacother. 99, 583–590. 10.1016/j.biopha.2018.01.067 29710456

[B59] Jiang M. Li Z. Zhu G. (2020). Immunological regulatory effect of flavonoid baicalin on innate immune toll-like receptors. Pharmacol. Res. 158, 104890. 10.1016/j.phrs.2020.104890 32389860

[B60] Jin R. Yang G. Li G. (2010). Inflammatory mechanisms in ischemic stroke: role of inflammatory cells. J. Leukoc. Biol. 87 (5), 779–789. 10.1189/jlb.1109766 20130219 PMC2858674

[B61] Jivad N. Rabiei Z. J. A. P. J. o. T. B. (2015). Review on herbal medicine on brain ischemia and reperfusion. Asian pac. J. Trop. Biomed. 5 (10), 789–795. 10.1016/j.apjtb.2015.07.015

[B62] Kaviarasi S. Yuba E. Harada A. Krishnan U. M. (2019). Emerging paradigms in nanotechnology for imaging and treatment of cerebral ischemia. J. Control Release 300, 22–45. 10.1016/j.jconrel.2019.02.031 30802476

[B63] Khaksar S. Bigdeli M. R. (2017). Anti-excitotoxic effects of cannabidiol are partly mediated by enhancement of NCX2 and NCX3 expression in animal model of cerebral ischemia. Eur. J. Pharmacol. 794, 270–279. 10.1016/j.ejphar.2016.11.011 27856160

[B64] Khoshnam S. E. Farbood Y. Fathi Moghaddam H. Sarkaki A. Badavi M. Khorsandi L. (2018). Vanillic acid attenuates cerebral hyperemia, blood-brain barrier disruption and anxiety-like behaviors in rats following transient bilateral common carotid occlusion and reperfusion. Metab. Brain Dis. 33 (3), 785–793. 10.1007/s11011-018-0187-5 29356980

[B65] Kovalska M. Kovalska L. Pavlikova M. Janickova M. Mikuskova K. Adamkov M. (2012). Intracellular signaling MAPK pathway after cerebral ischemia-reperfusion injury. Neurochem. Res. 37 (7), 1568–1577. 10.1007/s11064-012-0752-y 22431068

[B66] Kuranaga E. (2012). Beyond apoptosis: caspase regulatory mechanisms and functions *in vivo* . Genes. cells. 17 (2), 83–97. 10.1111/j.1365-2443.2011.01579.x 22244258

[B67] Lambertsen K. L. Biber K. Finsen B. (2012). Inflammatory cytokines in experimental and human stroke. J. Cereb. Blood Flow. Metab. 32 (9), 1677–1698. 10.1038/jcbfm.2012.88 22739623 PMC3434626

[B68] Leech T. Chattipakorn N. Chattipakorn S. C. (2019). The beneficial roles of metformin on the brain with cerebral ischaemia/reperfusion injury. Pharmacol. Res. 146, 104261. 10.1016/j.phrs.2019.104261 31170502

[B69] Li C. Jackson R. M. (2002). Reactive species mechanisms of cellular hypoxia-reoxygenation injury. Am. J. Physiol. Cell. Physiol. 282 (2), C227–C241. 10.1152/ajpcell.00112.2001 11788333

[B70] Li F. Mao Q. Wang J. Zhang X. Lv X. Wu B. (2022a). Salidroside inhibited cerebral ischemia/reperfusion-induced oxidative stress and apoptosis via Nrf2/Trx1 signaling pathway. Metab. Brain Dis. 37 (8), 2965–2978. 10.1007/s11011-022-01061-x 35976554

[B71] Li H. Xia Z. Chen Y. Qi D. Zheng H. (2018a). Mechanism and therapies of oxidative stress-mediated cell death in ischemia reperfusion injury. Oxid. Med. Cell. Longev. 2018, 2910643. 10.1155/2018/2910643 30034574 PMC6035842

[B72] Li L. Zhang D. Yao W. Wu Z. Cheng J. Ji Y. (2022b). Ligustrazine exerts neuroprotective effects via circ_0008146/miR-709/Cx3cr1 axis to inhibit cell apoptosis and inflammation after cerebral ischemia/reperfusion injury. Brain Res. Bull. 190, 244–255. 10.1016/j.brainresbull.2022.10.011 36244580

[B73] Li M. Li L. H. Qu Y. Z. Gao G. D. (2013). Astragaloside IV reduces cerebral edema post-ischemia/reperfusion correlating the suppression of MMP-9 and AQP4. Eur. J. Pharmacol. 715 (1-3), 189–195. 10.1016/j.ejphar.2013.05.022 23747593

[B74] Li M. Meng Z. Yu S. Li J. Wang Y. Yang W. (2022c). Baicalein ameliorates cerebral ischemia-reperfusion injury by inhibiting ferroptosis via regulating GPX4/ACSL4/ACSL3 axis. Chem. Biol. Interact. 366, 110137. 10.1016/j.cbi.2022.110137 36055377

[B75] Li P. Stetler R. A. Leak R. K. Shi Y. Li Y. Yu W. (2018b). Oxidative stress and DNA damage after cerebral ischemia: potential therapeutic targets to repair the genome and improve stroke recovery. Neuropharmacology 134 (Pt B), 208–217. 10.1016/j.neuropharm.2017.11.011 29128308 PMC5940593

[B76] Li Q. Wu J. Huang L. Zhao B. (2021). Ephedrine ameliorates cerebral ischemia injury via inhibiting NOD-like receptor pyrin domain 3 inflammasome activation through the Akt/GSK3β/NRF2 pathway. Hum. Exp. Toxicol. 40 (12_Suppl. l), S540–s552. 10.1177/09603271211052981 34715758

[B77] Li Y. Lin T. Y. Luo Y. Liu Q. Xiao W. Guo W. (2014). A smart and versatile theranostic nanomedicine platform based on nanoporphyrin. Nat. Commun. 5, 4712. 10.1038/ncomms5712 25158161 PMC4145614

[B78] Liang J. Cai J. Zhang Y. Xie M. Li X. Hu T. (2022). Cyclo-(Phe-Tyr) reduces cerebral ischemia/reperfusion-induced blood-brain barrier dysfunction through regulation of autophagy. Food Funct. 13 (23), 12278–12290. 10.1039/d2fo02367a 36345882

[B79] Lin J. Hao C. Gong Y. Zhang Y. Li Y. Feng Z. (2021). Effect of tetramethylpyrazine on neuroplasticity after transient focal cerebral ischemia reperfusion in rats. Evid. Based Complement. Altern. Med. 2021, 1587241. 10.1155/2021/1587241 PMC7834793 33531914

[B80] Lin M. Sun W. Gong W. Zhou Z. Ding Y. Hou Q. (2015). Methylophiopogonanone A protects against cerebral ischemia/reperfusion injury and attenuates blood-brain barrier disruption *in vitro* . PLoS One 10 (4), e0124558. 10.1371/journal.pone.0124558 25897666 PMC4405202

[B81] Liu D. M. Cao Z. X. Yan H. L. Li W. Yang F. Zhao W. J. (2020). A new abietane diterpenoid from Ajuga ovalifolia var. calantha induces human lung epithelial A549 cell apoptosis by inhibiting SHP2. Fitoterapia 141, 104484. 10.1016/j.fitote.2020.104484 31954180

[B82] Liu D. Q. Chen S. P. Sun J. Wang X. M. Chen N. Zhou Y. Q. (2019). Berberine protects against ischemia-reperfusion injury: a review of evidence from animal models and clinical studies. Pharmacol. Res. 148, 104385. 10.1016/j.phrs.2019.104385 31400402

[B83] Liu H. Xu Y. Wang X. Ren R. Zhu H. (2021). Engeletin protects against cerebral ischemia/reperfusion injury by modulating the VEGF/vasohibin and Ang-1/Tie-2 pathways. Braz J. Med. Biol. Res. 54 (10), e11028. 10.1590/1414-431X2020e11028 34287581 PMC8289342

[B84] Liu J. Chen Q. Jian Z. Xiong X. Shao L. Jin T. (2016a). Daphnetin protects against cerebral ischemia/reperfusion injury in mice via inhibition of TLR4/NF-κB signaling pathway. Biomed. Res. Int. 2016, 2816056. 10.1155/2016/2816056 28119924 PMC5227117

[B85] Liu P. Zhao H. Wang R. Wang P. Tao Z. Gao L. (2015). MicroRNA-424 protects against focal cerebral ischemia and reperfusion injury in mice by suppressing oxidative stress. Stroke 46 (2), 513–519. 10.1161/STROKEAHA.114.007482 25523055

[B86] Liu T. Liu M. Zhang T. Liu W. Xu H. Mu F. (2018). Z-Guggulsterone attenuates astrocytes-mediated neuroinflammation after ischemia by inhibiting toll-like receptor 4 pathway. J. Neurochem. 147 (6), 803–815. 10.1111/jnc.14583 30168601

[B87] Liu T. Xiang B. Guo D. Sun F. Wei R. Zhang G. (2016b). Morroniside promotes angiogenesis and further improves microvascular circulation after focal cerebral ischemia/reperfusion. Brain Res. Bull. 127, 111–118. 10.1016/j.brainresbull.2016.09.004 27614236

[B88] Liu X. Chen X. Zhu Y. Wang K. Wang Y. (2017). Effect of magnolol on cerebral injury and blood brain barrier dysfunction induced by ischemia-reperfusion *in vivo* and *in vitro* . Metab. Brain Dis. 32 (4), 1109–1118. 10.1007/s11011-017-0004-6 28378105

[B89] Liu Y. Zhu X. Tong X. Tan Z. (2022). Syringin protects against cerebral ischemia/reperfusion injury via inhibiting neuroinflammation and TLR4 signaling. Perfusion 37 (6), 562–569. 10.1177/02676591211007025 33832376

[B90] Long Y. Xiang Y. Liu S. Zhang Y. Wan J. Ci Z. (2022). Macrophage membrane modified baicalin liposomes improve brain targeting for alleviating cerebral ischemia reperfusion injury. Nanomedicine 43, 102547. 10.1016/j.nano.2022.102547 35292367

[B91] Lu L. Y. Liu Y. Gong Y. F. Zheng X. Y. (2019). A preliminary report: genistein attenuates cerebral ischemia injury in ovariectomized rats via regulation of the PI3K-Akt-mTOR pathway. Gen. Physiol. Biophys. 38 (5), 389–397. 10.4149/gpb_2019024 31595881

[B92] Lv H. Wang L. Shen J. Hao S. Ming A. Wang X. (2015). Salvianolic acid B attenuates apoptosis and inflammation via SIRT1 activation in experimental stroke rats. Brain Res. Bull. 115, 30–36. 10.1016/j.brainresbull.2015.05.002 25981395

[B93] Mahmood Q. Wang G. F. Wu G. Wang H. Zhou C. X. Yang H. Y. (2017). Salvianolic acid A inhibits calpain activation and eNOS uncoupling during focal cerebral ischemia in mice. Phytomedicine 25, 8–14. 10.1016/j.phymed.2016.12.004 28190474

[B94] Maleki S. N. Aboutaleb N. Souri F. (2018). Berberine confers neuroprotection in coping with focal cerebral ischemia by targeting inflammatory cytokines. J. Chem. Neuroanat. 87, 54–59. 10.1016/j.jchemneu.2017.04.008 28495517 PMC5812778

[B95] Mao Z. Tian L. Liu J. Wu Q. Wang N. Wang G. (2022). Ligustilide ameliorates hippocampal neuronal injury after cerebral ischemia reperfusion through activating PINK1/Parkin-dependent mitophagy. Phytomedicine 101, 154111. 10.1016/j.phymed.2022.154111 35512628

[B96] Meng X. Xie W. Xu Q. Liang T. Xu X. Sun G. (2018). Neuroprotective effects of radix scrophulariae on cerebral ischemia and reperfusion injury via MAPK pathways. Molecules 23 (9), 2401. 10.3390/molecules23092401 30235876 PMC6225418

[B97] Miao Y. Zhao S. Gao Y. Wang R. Wu Q. Wu H. (2016). Curcumin pretreatment attenuates inflammation and mitochondrial dysfunction in experimental stroke: the possible role of Sirt1 signaling. Brain Res. Bull. 121, 9–15. 10.1016/j.brainresbull.2015.11.019 26639783

[B98] Mirshekari Jahangiri H. Sarkaki A. Farbood Y. Dianat M. Goudarzi G. (2020). Gallic acid affects blood-brain barrier permeability, behaviors, hippocampus local EEG, and brain oxidative stress in ischemic rats exposed to dusty particulate matter. Environ. Sci. Pollut. Res. Int. 27 (5), 5281–5292. 10.1007/s11356-019-07076-9 31848951

[B99] Mohammadi S. Jafari B. Asgharian P. Martorell M. Sharifi-Rad J. (2020). Medicinal plants used in the treatment of malaria: a key emphasis to Artemisia, cinchona, cryptolepis, and tabebuia genera. Phytother. Res. 34 (7), 1556–1569. 10.1002/ptr.6628 32022345

[B100] Mollazadeh H. Cicero A. F. G. Blesso C. N. Pirro M. Majeed M. Sahebkar A. (2019). Immune modulation by curcumin: the role of interleukin-10. Crit. Rev. Food Sci. Nutr. 59 (1), 89–101. 10.1080/10408398.2017.1358139 28799796

[B101] Morris C. A. Duparc S. Borghini-Fuhrer I. Jung D. Shin C. S. Fleckenstein L. (2011). Review of the clinical pharmacokinetics of artesunate and its active metabolite dihydroartemisinin following intravenous, intramuscular, oral or rectal administration. Malar. J. 10, 263. 10.1186/1475-2875-10-263 21914160 PMC3180444

[B102] Nam J. E. Jo S. Y. Ahn C. W. Kim Y. S. (2020). Baicalin attenuates fibrogenic process in human renal proximal tubular cells (HK-2) exposed to diabetic milieu. Life Sci. 254, 117742. 10.1016/j.lfs.2020.117742 32360619

[B103] Ni G. X. Liang C. Wang J. Duan C. Q. Wang P. Wang Y. L. (2020). Astragaloside IV improves neurobehavior and promotes hippocampal neurogenesis in MCAO rats though BDNF-TrkB signaling pathway. Biomed. Pharmacother. 130, 110353. 10.1016/j.biopha.2020.110353 32682983

[B104] Ni H. Li J. Zheng J. Zhou B. (2022). Cardamonin attenuates cerebral ischemia/reperfusion injury by activating the HIF-1α/VEGFA pathway. Phytother. Res. 36 (4), 1736–1747. 10.1002/ptr.7409 35142404

[B105] Obeng E. (2021). Apoptosis (programmed cell death) and its signals - a review. Braz J. Biol. 81 (4), 1133–1143. 10.1590/1519-6984.228437 33111928

[B106] Ojo O. B. Amoo Z. A. Saliu I. O. Olaleye M. T. Farombi E. O. Akinmoladun A. C. (2019). Neurotherapeutic potential of kolaviron on neurotransmitter dysregulation, excitotoxicity, mitochondrial electron transport chain dysfunction and redox imbalance in 2-VO brain ischemia/reperfusion injury. Biomed. Pharmacother. 111, 859–872. 10.1016/j.biopha.2018.12.144 30841465

[B107] Peng T. Li S. Liu L. Yang C. Farhan M. Chen L. (2022). Artemisinin attenuated ischemic stroke induced cell apoptosis through activation of ERK1/2/CREB/BCL-2 signaling pathway *in vitro* and *in vivo* . Int. J. Biol. Sci. 18 (11), 4578–4594. 10.7150/ijbs.69892 35864966 PMC9295073

[B108] Perluigi M. Coccia R. Butterfield D. A. (2012). 4-Hydroxy-2-nonenal, a reactive product of lipid peroxidation, and neurodegenerative diseases: a toxic combination illuminated by redox proteomics studies. Antioxid. Redox Signal 17 (11), 1590–1609. 10.1089/ars.2011.4406 22114878 PMC3449441

[B109] Priyadarsini K. I. (2014). The chemistry of curcumin: from extraction to therapeutic agent. Molecules 19 (12), 20091–20112. 10.3390/molecules191220091 25470276 PMC6270789

[B110] Qin C. Zhou P. Wang L. Mamtilahun M. Li W. Zhang Z. (2019). Dl-3-N-butylphthalide attenuates ischemic reperfusion injury by improving the function of cerebral artery and circulation. J. Cereb. Blood Flow. Metab. 39 (10), 2011–2021. 10.1177/0271678X18776833 29762050 PMC6775578

[B111] Qiu J. Wang M. Zhang J. Cai Q. Lu D. Li Y. (2016). The neuroprotection of Sinomenine against ischemic stroke in mice by suppressing NLRP3 inflammasome via AMPK signaling. Int. Immunopharmacol. 40, 492–500. 10.1016/j.intimp.2016.09.024 27769021

[B112] Ran Y. Qie S. Gao F. Ding Z. Yang S. Tian G. (2021b). Baicalein ameliorates ischemic brain damage through suppressing proinflammatory microglia polarization via inhibiting the TLR4/NF-κB and STAT1 pathway. Brain Res. 1770, 147626. 10.1016/j.brainres.2021.147626 34418356

[B113] Ran Y. Su W. Gao F. Ding Z. Yang S. Ye L. (2021a). Curcumin ameliorates white matter injury after ischemic stroke by inhibiting microglia/macrophage pyroptosis through NF-κB suppression and NLRP3 inflammasome inhibition. Oxid. Med. Cell. Longev. 2021, 1552127. 10.1155/2021/1552127 34630845 PMC8497115

[B114] Rao J. Wu Y. Fan X. Yang S. Jiang L. Dong Z. (2022). Facilitating mitophagy via pink1/parkin2 signaling is essential for the neuroprotective effect of β-caryophyllene against CIR-induced neuronal injury. Brain Sci. 12 (7), 868. 10.3390/brainsci12070868 35884674 PMC9313355

[B115] Rosenberg G. A. Estrada E. Y. Dencoff J. E. (1998). Matrix metalloproteinases and TIMPs are associated with blood-brain barrier opening after reperfusion in rat brain. Stroke 29 (10), 2189–2195. 10.1161/01.str.29.10.2189 9756602

[B116] Roth G. A. Mensah G. A. Johnson C. O. Addolorato G. Ammirati E. Baddour L. M. (2020). Global burden of cardiovascular diseases and risk factors, 1990-2019: update from the GBD 2019 study. J. Am. Coll. Cardiol. 76 (25), 2982–3021. 10.1016/j.jacc.2020.11.010 33309175 PMC7755038

[B117] Sapkota A. Gaire B. P. Cho K. S. Jeon S. J. Kwon O. W. Jang D. S. (2017). Eupatilin exerts neuroprotective effects in mice with transient focal cerebral ischemia by reducing microglial activation. PLoS One 12 (2), e0171479. 10.1371/journal.pone.0171479 28178289 PMC5298292

[B118] Shang J. Jiao J. Yan M. Wang J. Li Q. Shabuerjiang L. (2023). Chrysin protects against cerebral ischemia-reperfusion injury in hippocampus via restraining oxidative stress and transition elements. Biomed. Pharmacother. 161, 114534. 10.1016/j.biopha.2023.114534 36933376

[B119] Shen H. Pei H. Zhai L. Guan Q. Wang G. (2022). Salvianolic acid C improves cerebral ischemia reperfusion injury through suppressing microglial cell M1 polarization and promoting cerebral angiogenesis. Int. Immunopharmacol. 110, 109021. 10.1016/j.intimp.2022.109021 35810493

[B120] Shi C. Li J. Li J. (2021). Ephedrine attenuates cerebral ischemia/reperfusion injury in rats through NF-κB signaling pathway. Hum. Exp. Toxicol. 40 (6), 994–1002. 10.1177/0960327120975456 33307823

[B121] Shi J. Xiao H. Li J. Zhang J. Li Y. Zhang J. (2018). Wild-type p53-modulated autophagy and autophagic fibroblast apoptosis inhibit hypertrophic scar formation. Lab. Investig. 98 (11), 1423–1437. 10.1038/s41374-018-0099-3 30089855

[B122] Shi M. Wang J. Bi F. Bai Z. (2022). Diosmetin alleviates cerebral ischemia-reperfusion injury through Keap1-mediated Nrf2/ARE signaling pathway activation and NLRP3 inflammasome inhibition. Environ. Toxicol. 37 (6), 1529–1542. 10.1002/tox.23504 35191607

[B123] Shin E. J. Tran H. Q. Nguyen P. T. Jeong J. H. Nah S. Y. Jang C. G. (2018). Role of mitochondria in methamphetamine-induced dopaminergic neurotoxicity: involvement in oxidative stress, neuroinflammation, and pro-apoptosis-A review. Neurochem. Res. 43 (1), 66–78. 10.1007/s11064-017-2318-5 28589520

[B124] Shou J. W. Li X. X. Tang Y. S. Lim-Ho Kong B. Wu H. Y. Xiao M. J. (2022). Novel mechanistic insight on the neuroprotective effect of berberine: the role of PPARδ for antioxidant action. Free Radic. Biol. Med. 181, 62–71. 10.1016/j.freeradbiomed.2022.01.022 35093536

[B125] Sies H. (2015). Oxidative stress: a concept in redox biology and medicine. Redox Biol. 4, 180–183. 10.1016/j.redox.2015.01.002 25588755 PMC4309861

[B126] Singh S. K. Srivastav S. Castellani R. J. Plascencia-Villa G. Perry G. (2019). Neuroprotective and antioxidant effect of Ginkgo biloba Extract against AD and other neurological disorders. Neurotherapeutics 16 (3), 666–674. 10.1007/s13311-019-00767-8 31376068 PMC6694352

[B127] Slevin M. Kumar P. Gaffney J. Kumar S. Krupinski J. (2006). Can angiogenesis be exploited to improve stroke outcome? Mechanisms and therapeutic potential. Clin. Sci. (Lond) 111 (3), 171–183. 10.1042/CS20060049 16901264

[B128] Song X. Gong Z. Liu K. Kou J. Liu B. Liu K. (2020). Baicalin combats glutamate excitotoxicity via protecting glutamine synthetase from ROS-induced 20S proteasomal degradation. Redox Biol. 34, 101559. 10.1016/j.redox.2020.101559 32473460 PMC7260594

[B129] Stoll G. Kleinschnitz C. Nieswandt B. (2008). Molecular mechanisms of thrombus formation in ischemic stroke: novel insights and targets for treatment. Blood 112 (9), 3555–3562. 10.1182/blood-2008-04-144758 18676880

[B130] Su L. J. Ren Y. C. Chen Z. Ma H. F. Zheng F. Li F. (2022). Ginsenoside Rb1 improves brain, lung, and intestinal barrier damage in middle cerebral artery occlusion/reperfusion (MCAO/R) micevia the PPARγ signaling pathway. Chin. J. Nat. Med. 20 (8), 561–571. 10.1016/S1875-5364(22)60204-8 36031228

[B131] Sun F. L. Wang W. Cheng H. Wang Y. Li L. Xue J. L. (2014). Morroniside improves microvascular functional integrity of the neurovascular unit after cerebral ischemia. PLoS One 9 (6), e101194. 10.1371/journal.pone.0101194 24979385 PMC4076313

[B132] Sun X. Wang D. Zhang T. Lu X. Duan F. Ju L. (2020). Eugenol attenuates cerebral ischemia-reperfusion injury by enhancing autophagy via AMPK-mTOR-P70S6K pathway. Front. Pharmacol. 11, 84. 10.3389/fphar.2020.00084 32153404 PMC7047211

[B133] Thakur S. Riyaz B. Patil A. Kaur A. Kapoor B. Mishra V. (2018). Novel drug delivery systems for NSAIDs in management of rheumatoid arthritis: an overview. Biomed. Pharmacother. 106, 1011–1023. 10.1016/j.biopha.2018.07.027 30119166

[B134] Thiebaut A. M. Hedou E. Marciniak S. J. Vivien D. Roussel B. D. (2019). Proteostasis during cerebral ischemia. Front. Neurosci. 13, 637. 10.3389/fnins.2019.00637 31275110 PMC6594416

[B135] Tian Z. Tang C. Wang Z. (2019). Neuroprotective effect of ginkgetin in experimental cerebral ischemia/reperfusion via apoptosis inhibition and PI3K/Akt/mTOR signaling pathway activation. J. Cell. Biochem. 120 (10), 18487–18495. 10.1002/jcb.29169 31265179

[B136] Tsakiri N. Kimber I. Rothwell N. J. Pinteaux E. (2008). Interleukin-1-induced interleukin-6 synthesis is mediated by the neutral sphingomyelinase/Src kinase pathway in neurones. Br. J. Pharmacol. 153 (4), 775–783. 10.1038/sj.bjp.0707610 18059318 PMC2259207

[B137] Uzdensky A. B. (2019). Apoptosis regulation in the penumbra after ischemic stroke: expression of pro- and antiapoptotic proteins. Apoptosis 24 (9-10), 687–702. 10.1007/s10495-019-01556-6 31256300

[B138] Vanden Hoek T. L. Qin Y. Wojcik K. Li C. Q. Shao Z. H. Anderson T. (2003). Reperfusion, not simulated ischemia, initiates intrinsic apoptosis injury in chick cardiomyocytes. Am. J. Physiol. Heart Circ. Physiol. 284 (1), H141–H150. 10.1152/ajpheart.00132.2002 12388298 PMC7359634

[B139] Wang G. Guo H. Wang X. (2019a). Platycodin D protects cortical neurons against oxygen-glucose deprivation/reperfusion in neonatal hypoxic-ischemic encephalopathy. J. Cell. Biochem. 120 (8), 14028–14034. 10.1002/jcb.28677 30945345

[B140] Wang H. L. Zhou Q. H. Xu M. B. Zhou X. L. Zheng G. Q. (2017). Astragaloside IV for experimental focal cerebral ischemia: preclinical evidence and possible mechanisms. Oxid. Med. Cell. Longev. 2017, 8424326. 10.1155/2017/8424326 28303172 PMC5337886

[B141] Wang J. Guo M. Ma R. Wu M. Zhang Y. (2020a). Tetrandrine alleviates cerebral ischemia/reperfusion injury by suppressing NLRP3 inflammasome activation via Sirt-1. PeerJ 8, e9042. 10.7717/peerj.9042 32419986 PMC7211409

[B142] Wang J. Hu J. Chen X. Lei X. Feng H. Wan F. (2021a). Traditional Chinese medicine monomers: novel strategy for endogenous neural stem cells activation after stroke. Front. Cell. Neurosci. 15, 628115. 10.3389/fncel.2021.628115 33716673 PMC7952516

[B143] Wang J. J. Cui P. (2013). Neohesperidin attenuates cerebral ischemia-reperfusion injury via inhibiting the apoptotic pathway and activating the Akt/Nrf2/HO-1 pathway. J. Asian Nat. Prod. Res. 15 (9), 1023–1037. 10.1080/10286020.2013.827176 23952707

[B144] Wang L. Zhao H. Zhai Z. Z. Qu L. X. (2018). Protective effect and mechanism of ginsenoside Rg1 in cerebral ischaemia-reperfusion injury in mice. Biomed. Pharmacother. 99, 876–882. 10.1016/j.biopha.2018.01.136 29710487

[B145] Wang W. Xu J. (2020). Curcumin attenuates cerebral ischemia-reperfusion injury through regulating mitophagy and preserving mitochondrial function. Curr. Neurovasc Res. 17 (2), 113–122. 10.2174/1567202617666200225122620 32096742

[B146] Wang X. Yang G. (2020). Saikosaponin A attenuates neural injury caused by ischemia/reperfusion. Transl. Neurosci. 11 (1), 227–235. 10.1515/tnsci-2020-0129 33335763 PMC7712316

[B147] Wang Y. Chen G. Yu X. Li Y. Zhang L. He Z. (2016). Salvianolic acid B ameliorates cerebral ischemia/reperfusion injury through inhibiting TLR4/MyD88 signaling pathway. Inflammation 39 (4), 1503–1513. 10.1007/s10753-016-0384-5 27255374

[B148] Wang Y. K. Deng F. Miao J. Xie H. Feng J. C. (2015b). Neuroprotection by carbenoxolone against ischemia injury involves PI3K/Akt pathway. Clin. Lab. 61 (10), 1561–1568. 10.7754/clin.lab.2015.150215 26642720

[B149] Wang Y. Meng C. Zhang J. Wu J. Zhao J. (2019b). Inhibition of GSK-3β alleviates cerebral ischemia/reperfusion injury in rats by suppressing NLRP3 inflammasome activation through autophagy. Int. Immunopharmacol. 68, 234–241. 10.1016/j.intimp.2018.12.042 30703695

[B150] Wang Y. Zhang S. Ni H. Zhang Y. Yan X. Gao Y. (2021b). Autophagy is involved in the neuroprotective effect of nicotiflorin. J. Ethnopharmacol. 278, 114279. 10.1016/j.jep.2021.114279 34087402

[B151] Wang Y. Zhen Y. Wu X. Jiang Q. Li X. Chen Z. (2015a). Vitexin protects brain against ischemia/reperfusion injury via modulating mitogen-activated protein kinase and apoptosis signaling in mice. Phytomedicine 22 (3), 379–384. 10.1016/j.phymed.2015.01.009 25837275

[B152] Wang Z. Ren W. Zhao F. Han Y. Liu C. Jia K. (2020b). Curcumin amends Ca(2+) dysregulation in microglia by suppressing the activation of P2X7 receptor. Mol. Cell. Biochem. 465 (1-2), 65–73. 10.1007/s11010-019-03668-8 31894530

[B153] Wu D. Shi J. Elmadhoun O. Duan Y. Zhang J. (2017b). Dihydrocapsaicin (DHC) enhances the hypothermia-induced neuroprotection following ischemic stroke via PI3K/Akt regulation in rat. Brain Res. 1671, 18–25. 10.1016/j.brainres.2017.06.029 28684048

[B154] Wu G. Zhu L. Yuan X. Chen H. Xiong R. Zhang S. (2017a). Britanin ameliorates cerebral ischemia-reperfusion injury by inducing the Nrf2 protective pathway. Antioxid. Redox Signal 27 (11), 754–768. 10.1089/ars.2016.6885 28186440

[B155] Wu P. F. Zhang Z. Wang F. Chen J. g. (2010). Natural compounds from traditional medicinal herbs in the treatment of cerebral ischemia/reperfusion injury. Acta Pharmacol. Sin. 31 (12), 1523–1531. 10.1038/aps.2010.186 21127495 PMC4002952

[B156] Wu Q. Liu J. Mao Z. Tian L. Wang N. Wang G. (2022). Ligustilide attenuates ischemic stroke injury by promoting Drp1-mediated mitochondrial fission via activation of AMPK. Phytomedicine 95, 153884. 10.1016/j.phymed.2021.153884 34929562

[B157] Wu S. Guo T. Qi W. Li Y. Gu J. Liu C. (2021b). Curcumin ameliorates ischemic stroke injury in rats by protecting the integrity of the blood-brain barrier. Exp. Ther. Med. 22 (1), 783. 10.3892/etm.2021.10215 34055082 PMC8145684

[B158] Wu W. Qiu C. Feng X. Tao X. Zhu Q. Chen Z. (2020). Protective effect of paeoniflorin on acute cerebral infarction in rats. Curr. Pharm. Biotechnol. 21 (8), 702–709. 10.2174/1389201021666191224151634 31884927

[B159] Wu Y. Fan L. Wang Y. Ding J. Wang R. (2021a). Isorhamnetin alleviates high glucose-aggravated inflammatory response and apoptosis in oxygen-glucose deprivation and reoxygenation-induced HT22 hippocampal neurons through akt/SIRT1/nrf2/HO-1 signaling pathway. Inflammation 44 (5), 1993–2005. 10.1007/s10753-021-01476-1 33999329

[B160] Xiao L. Dai Z. Tang W. Liu C. Tang B. (2021). Astragaloside IV alleviates cerebral ischemia-reperfusion injury through NLRP3 inflammasome-mediated pyroptosis inhibition via activating Nrf2. Oxid. Med. Cell. Longev. 2021, 9925561. 10.1155/2021/9925561 35003524 PMC8739174

[B161] Xie C. L. Li J. H. Wang W. W. Zheng G. Q. Wang L. X. (2015). Neuroprotective effect of ginsenoside-Rg1 on cerebral ischemia/reperfusion injury in rats by downregulating protease-activated receptor-1 expression. Life Sci. 121, 145–151. 10.1016/j.lfs.2014.12.002 25498890

[B162] Xie W. Wulin H. Shao G. Wei L. Qi R. Ma B. (2020). Polygalasaponin F inhibits neuronal apoptosis induced by oxygen-glucose deprivation and reoxygenation through the PI3K/Akt pathway. Basic Clin. Pharmacol. Toxicol. 127 (3), 196–204. 10.1111/bcpt.13408 32237267

[B163] Xie W. Zhou P. Sun Y. Meng X. Dai Z. Sun G. (2018). Protective effects and target network analysis of ginsenoside Rg1 in cerebral ischemia and reperfusion injury: a comprehensive overview of experimental studies. Cells 7 (12), 270. 10.3390/cells7120270 30545139 PMC6316103

[B164] Xie Z. Shi M. Zhang C. Zhao H. Hui H. Zhao G. (2016). Ginsenoside Rd protects against cerebral ischemia-reperfusion injury via decreasing the expression of the NMDA receptor 2B subunit and its phosphorylated product. Neurochem. Res. 41 (8), 2149–2159. 10.1007/s11064-016-1930-0 27165636

[B165] Xu H. Nie B. Liu L. Zhang C. Zhang Z. Xu M. (2019). Curcumin prevents brain damage and cognitive dysfunction during ischemic-reperfusion through the regulation of miR-7-5p. Curr. Neurovasc Res. 16 (5), 441–454. 10.2174/1567202616666191029113633 31660818

[B166] Xu Z. Liu W. Huang H. (2020). Astragaloside IV alleviates cerebral ischemia-reperfusion injury by activating the janus kinase 2 and signal transducer and activator of transcription 3 signaling pathway. Pharmacology 105 (3-4), 181–189. 10.1159/000503361 31825924

[B167] Yaidikar L. Thakur S. (2015). Arjunolic acid, a pentacyclic triterpenoidal saponin of Terminalia arjuna bark protects neurons from oxidative stress associated damage in focal cerebral ischemia and reperfusion. Pharmacol. Rep. 67 (5), 890–895. 10.1016/j.pharep.2015.02.003 26398381

[B168] Yang C. Hawkins K. E. Doré S. Candelario-Jalil E. (2019a). Neuroinflammatory mechanisms of blood-brain barrier damage in ischemic stroke. Am. J. Physiol. Cell. Physiol. 316 (2), C135–c153. 10.1152/ajpcell.00136.2018 30379577 PMC6397344

[B169] Yang F. Ma Q. Matsabisa M. G. Chabalala H. Braga F. C. Tang M. (2020). Panax notoginseng for cerebral ischemia: a systematic review. Am. J. Chin. Med. 48 (6), 1331–1351. 10.1142/S0192415X20500652 32907361

[B170] Yang H. Liu Q. Zong S. B. Li L. Xu Z. L. (2021). Neuroprotective effects of Ginkgolide B in focal cerebral ischemia through selective activation of prostaglandin E2 receptor EP4 and the downstream transactivation of epidermal growth factor receptor. Phytother. Res. 35, 2727–2744. 10.1002/ptr.7018 33452698

[B171] Yang J. L. Mukda S. Chen S. D. (2018). Diverse roles of mitochondria in ischemic stroke. Redox Biol. 16, 263–275. 10.1016/j.redox.2018.03.002 29549824 PMC5854930

[B172] Yang R. Shen Y. J. Chen M. Zhao J. Y. Chen S. H. Zhang W. (2022b). Quercetin attenuates ischemia reperfusion injury by protecting the blood-brain barrier through Sirt1 in MCAO rats. J. Asian Nat. Prod. Res. 24 (3), 278–289. 10.1080/10286020.2021.1949302 34292112

[B173] Yang S. Wang H. Yang Y. Wang R. Wang Y. Wu C. (2019b). Baicalein administered in the subacute phase ameliorates ischemia-reperfusion-induced brain injury by reducing neuroinflammation and neuronal damage. Biomed. Pharmacother. 117, 109102. 10.1016/j.biopha.2019.109102 31228802

[B174] Yang Y. He B. Zhang X. Yang R. Xia X. Chen L. (2022a). Geraniin protects against cerebral ischemia/reperfusion injury by suppressing oxidative stress and neuronal apoptosis via regulation of the Nrf2/HO-1 pathway. Oxid. Med. Cell. Longev. 2022, 2152746. 10.1155/2022/2152746 35222793 PMC8881129

[B175] Yao Y. Hu S. Zhang C. Zhou Q. Wang H. Yang Y. (2022). Ginsenoside Rd attenuates cerebral ischemia/reperfusion injury by exerting an anti-pyroptotic effect via the miR-139-5p/FoxO1/Keap1/Nrf2 axis. Int. Immunopharmacol. 105, 108582. 10.1016/j.intimp.2022.108582 35124564

[B176] Yu L. Duan Y. Zhao Z. He W. Xia M. Zhang Q. (2018). Hydroxysafflor yellow A (hsya) improves learning and memory in cerebral ischemia reperfusion-injured rats via recovering synaptic plasticity in the Hippocampus. Front. Cell. Neurosci. 12, 371. 10.3389/fncel.2018.00371 30405354 PMC6200869

[B177] Zeng M. Zhang R. Yang Q. Guo L. Zhang X. Yu B. (2022). Pharmacological therapy to cerebral ischemia-reperfusion injury: focus on saponins. Biomed. Pharmacother. 155, 113696. 10.1016/j.biopha.2022.113696 36116247

[B178] Zeng X. S. Zhou X. S. Luo F. C. Jia J. J. Qi L. Yang Z. X. (2014). Comparative analysis of the neuroprotective effects of ginsenosides Rg1 and Rb1 extracted from Panax notoginseng against cerebral ischemia. Can. J. Physiol. Pharmacol. 92 (2), 102–108. 10.1139/cjpp-2013-0274 24502632

[B179] Zhang J. F. Shi L. L. Zhang L. Zhao Z. H. Liang F. Xu X. (2016b). MicroRNA-25 negatively regulates cerebral ischemia/reperfusion injury-induced cell apoptosis through fas/FasL pathway. J. Mol. Neurosci. 58 (4), 507–516. 10.1007/s12031-016-0712-0 26768135

[B180] Zhang J. Liu M. Huang M. Chen M. Zhang D. Luo L. (2019b). Ginsenoside F1 promotes angiogenesis by activating the IGF-1/IGF1R pathway. Pharmacol. Res. 144, 292–305. 10.1016/j.phrs.2019.04.021 31048033

[B181] Zhang J. Wu C. Gao L. Du G. Qin X. (2020d). Astragaloside IV derived from Astragalus membranaceus: a research review on the pharmacological effects. Adv. Pharmacol. 87, 89–112. 10.1016/bs.apha.2019.08.002 32089240

[B182] Zhang K. Liu Q. Luo L. Feng X. Hu Q. Fan X. (2021a). Neuroprotective effect of alpha-asarone on the rats model of cerebral ischemia-reperfusion stroke via ameliorating glial activation and autophagy. Neuroscience 473, 130–141. 10.1016/j.neuroscience.2021.08.006 34416342

[B183] Zhang K. Yang Y. Ge H. Wang J. Chen X. Lei X. (2020e). Artesunate promotes the proliferation of neural stem/progenitor cells and alleviates Ischemia-reperfusion Injury through PI3K/Akt/FOXO-3a/p27(kip1) signaling pathway. Aging (Albany NY) 12 (9), 8029–8048. 10.18632/aging.103121 32379706 PMC7244066

[B184] Zhang Q. Bian H. Guo L. Zhu H. (2016a). Berberine preconditioning protects neurons against ischemia via sphingosine-1-phosphate and hypoxia-inducible factor-1[formula: see text]. Am. J. Chin. Med. 44 (5), 927–941. 10.1142/S0192415X16500518 27430910

[B185] Zhang Q. Bian H. Guo L. Zhu H. (2016c). Pharmacologic preconditioning with berberine attenuating ischemia-induced apoptosis and promoting autophagy in neuron. Am. J. Transl. Res. 8 (2), 1197–1207. 27158406 PMC4846963

[B186] Zhang Q. Yao M. Qi J. Song R. Wang L. Li J. (2023). Puerarin inhibited oxidative stress and alleviated cerebral ischemia-reperfusion injury through PI3K/Akt/Nrf2 signaling pathway. Front. Pharmacol. 14, 1134380. 10.3389/fphar.2023.1134380 37284311 PMC10240043

[B187] Zhang S. Jiang L. Che F. Lu Y. Xie Z. Wang H. (2017). Arctigenin attenuates ischemic stroke via SIRT1-dependent inhibition of NLRP3 inflammasome. Biochem. Biophys. Res. Commun. 493 (1), 821–826. 10.1016/j.bbrc.2017.08.062 28888980

[B188] Zhang T. Li Z. Qin Z. Cao Y. Shan T. Fang Y. (2021b). Neuroprotection of Chikusetsu saponin V on transient focal cerebral ischemia/reperfusion and the underlying mechanism. Phytomedicine 84, 153516. 10.1016/j.phymed.2021.153516 33639592

[B189] Zhang W. Song J. Li W. Kong D. Liang Y. Zhao X. (2020b). Salvianolic acid D alleviates cerebral ischemia-reperfusion injury by suppressing the cytoplasmic translocation and release of HMGB1-triggered NF-κB activation to inhibit inflammatory response. Mediat. Inflamm. 2020, 9049614. 10.1155/2020/9049614 PMC7204335 32410871

[B190] Zhang X. Wang L. Guo D. Y. Zhang J. M. Chen Y. G. (2020a). Analysis of clinical efficacy of traditional Chinese medicine in recovery stage of stroke: a systematic review and meta-analysis. Cardiovasc Ther. 2020, 7172052. 10.1155/2020/7172052 33042224 PMC7528130

[B191] Zhang X. Xue Z. Zhu S. Guo Y. Zhang Y. Dou J. (2022). Diosgenin revealed potential effect against cerebral ischemia reperfusion through HIKESHI/HSP70/NF-κB anti-inflammatory axis. Phytomedicine 99, 153991. 10.1016/j.phymed.2022.153991 35217435

[B192] Zhang Y. Jin X. F. Zhou X. H. Dong X. H. Yu W. T. (2019a). The role of astragaloside IV against cerebral ischemia/reperfusion injury: suppression of apoptosis via promotion of P62-LC3-autophagy. Molecules 24 (9), 1838. 10.3390/molecules24091838 31086091 PMC6539971

[B193] Zhang Y. Tian Z. Wan H. Liu W. Kong F. Ma G. (2020c). Deltonin ameliorates cerebral ischemia/reperfusion injury in correlation with modulation of autophagy and inflammation. Neuropsychiatr. Dis. Treat. 16, 871–879. 10.2147/NDT.S227988 32280228 PMC7127787

[B194] Zhao Y. Shi X. Wang J. Mang J. Xu Z. (2021). Betulinic acid ameliorates cerebral injury in middle cerebral artery occlusion rats through regulating autophagy. ACS Chem. Neurosci. 12 (15), 2829–2837. 10.1021/acschemneuro.1c00198 34296845

[B195] Zheng J. Liao Y. Xu Y. Mo Z. (2022). Icariin attenuates ischaemic stroke through suppressing inflammation mediated by endoplasmic reticulum stress signalling pathway in rats. Clin. Exp. Pharmacol. Physiol. 49 (7), 719–730. 10.1111/1440-1681.13645 35451526

[B196] Zheng T. Jiang H. Jin R. Zhao Y. Bai Y. Xu H. (2019). Ginsenoside Rg1 attenuates protein aggregation and inflammatory response following cerebral ischemia and reperfusion injury. Eur. J. Pharmacol. 853, 65–73. 10.1016/j.ejphar.2019.02.018 30794781

[B197] Zheng W. X. He W. Q. Zhang Q. R. Jia J. X. Zhao S. Wu F. J. (2021). Baicalin inhibits NLRP3 inflammasome activity via the AMPK signaling pathway to alleviate cerebral ischemia-reperfusion injury. Inflammation 44 (5), 2091–2105. 10.1007/s10753-021-01486-z 34080089

[B198] Zhong Z. F. Han J. Zhang J. Z. Xiao Q. Chen J. Y. Zhang K. (2019). Neuroprotective effects of salidroside on cerebral ischemia/reperfusion-induced behavioral impairment involves the dopaminergic system. Front. Pharmacol. 10, 1433. 10.3389/fphar.2019.01433 31920641 PMC6923222

[B199] Zhou F. Wang L. Liu P. Hu W. Zhu X. Shen H. (2014). Puerarin protects brain tissue against cerebral ischemia/reperfusion injury by inhibiting the inflammatory response. Neural Regen. Res. 9 (23), 2074–2080. 10.4103/1673-5374.147934 25657724 PMC4316472

[B200] Zhou K. Chen J. Wu J. Wu Q. Jia C. Xu Y. X. Z. (2019). Atractylenolide III ameliorates cerebral ischemic injury and neuroinflammation associated with inhibiting JAK2/STAT3/Drp1-dependent mitochondrial fission in microglia. Phytomedicine 59, 152922. 10.1016/j.phymed.2019.152922 30981186

[B201] Zhou L. Zuo Z. Chow M. S. (2005). Danshen: an overview of its chemistry, pharmacology, pharmacokinetics, and clinical use. J. Clin. Pharmacol. 45 (12), 1345–1359. 10.1177/0091270005282630 16291709

[B202] Zhou X. M. Cao Y. L. Dou D. Q. (2006). Protective effect of ginsenoside-Re against cerebral ischemia/reperfusion damage in rats. Biol. Pharm. Bull. 29 (12), 2502–2505. 10.1248/bpb.29.2502 17142990

[B203] Zhou X. Q. Zeng X. N. Kong H. Sun X. L. (2008). Neuroprotective effects of berberine on stroke models *in vitro* and *in vivo* . Neurosci. Lett. 447 (1), 31–36. 10.1016/j.neulet.2008.09.064 18838103

[B204] Zhu J. Wang L. Zhang J. (2020). Galuteolin inhibited autophagy for neuroprotection against transient focal cerebral ischemia in rats. Neuromolecular Med. 22 (4), 493–502. 10.1007/s12017-020-08606-2 33085067

[B205] Zhu T. Xie W. J. Wang L. Jin X. B. Meng X. B. Sun G. B. (2021). Notoginsenoside R1 activates the NAMPT-NAD(+)-SIRT1 cascade to promote postischemic angiogenesis by modulating Notch signaling. Biomed. Pharmacother. 140, 111693. 10.1016/j.biopha.2021.111693 34029951

[B206] Zuo S. Li Q. Zhang X. Hu R. Hu S. (2017). Artesunate protected blood-brain barrier via sphingosine 1 phosphate receptor 1/phosphatidylinositol 3 kinase pathway after subarachnoid hemorrhage in rats. Mol. Neurobiol. 54 (2), 1213–1228. 10.1007/s12035-016-9732-6 26820677

